# Integration of Network Pharmacology and Molecular Docking Together with an In Vitro Nitric Oxide Inhibition for the Insight for Antipyretic Effects of Benjalokawichian, the Thai Traditional Polyherbal Remedy

**DOI:** 10.3390/ijms27062697

**Published:** 2026-03-16

**Authors:** Chinnaphat Chaloemram, Ruchilak Rattarom, Anake Kijjoa, Somsak Nualkaew

**Affiliations:** 1 Doctor of Philosophy in Pharmacy Program, Faculty of Pharmacy, Mahasarakham University, Kantharawichai 44150, Maha Sarakham, Thailand; chinnaphat.med@gmail.com; 2 Pharmaceutical Chemistry and Natural Product Research Unit, Faculty of Pharmacy, Mahasarakham University, Kantharawichai 44150, Maha Sarakham, Thailand; rujiluk.r@msu.ac.th; 3 School of Medicine and Biomedical Sciences Abel Salazar (ICBAS) and CIIMAR, Universidade do Porto, Rua de Jorge Viterbo Ferreira 228, 4050-313 Porto, Portugal; ankijjoa@icbas.up.pt

**Keywords:** Benjalokawichian, Ha-Rak, toxic fever, network pharmacology, molecular docking, NO inhibition, anti-inflammatory

## Abstract

Benjalokawichian (BLW) is a classic antipyretic polyherbal remedy used in Thai traditional medicine (TTM) to reduce toxic fever (TF). This study aimed to shed light on the mechanisms of action and identify bioactive components of BLW responsible for TF treatment. The methods that combine network pharmacology, molecular docking, and the inhibition of nitric oxide (NO) production in LPS-induced RAW 264.7 were employed for these objectives. Network pharmacology served as a means to identify 15 potential bioactive compounds, 88 possible therapeutic targets, and 4 hub genes related to BLW. Among the significant targets, TNF, PTGS2, STAT3, and NFKB1 were closely linked to the metabolic pathways of phenylalanine, arachidonic acid, and tyrosine, which are vital in managing infections, inflammation, proliferation, and apoptosis in the TF microenvironment. Additionally, molecular docking analysis indicated that core compounds displayed strong binding affinities for the key targets, with binding energies ranging between −4.5 and −11.1 kcal/mol. The in vitro assay demonstrated that BLW extract significantly inhibited NO production in LPS-activated RAW 264.7 macrophages, presenting an IC_50_ value of 69.10 μg/mL, and no cytotoxic effects on RAW 264.7 macrophages. Furthermore, the biomarker compounds of BLW extract, viz., perforatic acid and peucenin-7-methyl ether were found to decrease NO production in a dose-dependent manner. In summary, this research indicates that BLW provides therapeutic benefits for TF via a complex interplay of different compounds, targets, and pathways. These findings serve as a foundation for further research into the mechanisms of action of a polyherbal remedy toward TF to provide scientific evidences for its clinical use.

## 1. Introduction

Fever is recognized as a fundamental host defense mechanism and a hallmark clinical manifestation of inflammatory responses. The febrile process is orchestrated by a complex interplay of endogenous and exogenous pyrogens, and often serves as a primary indicator for numerous pathological states, including microbial infections. These stimuli trigger biosynthesis and systemic release of diverse inflammatory mediators from both immune cells, such as macrophages and leukocytes, and non-immune lineages like endothelial cells [1,2,3]. At the molecular level, a critical phase of thermoregulatory adjustment involves the induction of the arachidonic acid metabolic pathway within the preoptic area and anterior hypothalamus (POAH). Specifically, pyrogenic cytokines, most notably interleukin-1β (IL1B), interleukin-6 (IL6), tumor necrosis factor-α (TNF), and interferons (IFNs), upregulate cyclooxygenase-2 (COX-2). This enzymatic activation results in the localized accumulation of prostaglandin E2 (PGE_2_), which subsequently resets the hypothalamic thermostat to elevate core body temperature [2,3,4,5,6]. While mild and self-limiting pyrexia generally requires minimal clinical intervention, severe febrile episodes present significant therapeutic challenges and risks. Severe fever is typically characterized by a sustained temperature of 40 °C or higher, or when accompanied by alarming neurological or respiratory symptoms such as nuchal rigidity, intense cephalalgia, or acute dyspnea [7]. Such severe cases often reflect profound systemic inflammation marked by a pathological surge in circulating pro-inflammatory cytokines and chemokines. This dysregulated phenomenon, frequently known as a cytokine storm, can precipitate acute respiratory distress syndrome (ARDS) and systemic multi-organ dysfunction, potentially leading to fatal outcomes if not managed with timely pharmacological intervention [8].

Toxic fever (TF) or Khai Phit is documented in the Taksila scriptures, which detail the outbreak and treatment of various fevers using principles of Thai traditional medicine (TTM) [9]. Potential signs of TF include a sudden high fever, accompanied by symptoms such as red eyes, cold extremities, headaches, alternating chills and heat, pain, stiff tongue and jaw, labored breathing, dry mouth, drowsiness, unconsciousness, delirium, and rashes appearing on the body, for example scarlet fever, roseola infantum measles, rubella, herpes zoster, erysipelas, and typhoid fever [10].

The Thai polyherbal remedy, Benjalokawichian (BLW), frequently referred to as Ha-Rak or the “Five Roots” remedy, is a well-known antipyretic preparation of TTM. Recognized for its therapeutic value, BLW was included in the National List of Herbal Medicinal Products of Thailand in 2006 and remains a standard prescription for the management of febrile illnesses. According to the Taksila scriptures, this traditional remedy is specifically indicated for alleviating toxic fever associated with inflammatory pathologies as well as for treatment of inflammatory and allergic dermatological conditions. BLW consists of equal proportions of roots of five plants, i.e., *Capparis micracantha* DC., *Clerodendrum indicum* (L.) Kuntze, *Ficus racemosa* L., *Harrisonia perforata* (Blanco) Merr., and *Tiliacora triandra* (Colebr.) Diels [11,12]. The traditional method for preparing BLW includes simmering equal portions (1–2 cm in length) of air-dried roots from the five plants in water (1:5 *w*/*v*) in a clay pot for 30 min, then allowing it to cool to ambient temperature and subsequently filtering it through cheesecloth to obtain a decoction. For adult patients, the traditional regimen consists of one glass taken three times per day before meals upon the onset of symptoms. Dosages for pediatric patients are calibrated based on the age of the child [9]. In accordance with the 2023 National List of Essential Herbal Medicines, BLW is also commercially available in modern dosage forms, including powders, tablets, and capsules. The current clinical guidelines suggest a dosage of 1 to 1.5 g per administration for adults. For children between 6 and 12 years of age, the recommended dose ranges from 0.5 to 1 g, to be taken three times daily before meals as symptoms persist [13].

Network pharmacology represents a multidimensional methodology that combines bioinformatics, cheminformatics, and systems biology to elucidate the intricate relationships among bioactive constituents, molecular targets, and disease-related signaling pathways. This strategy has gained more traction in the scientific community for its ability to predict potential binding targets for diverse chemical compounds, establish comprehensive interaction networks involving compounds, targets, and diseases, and pinpoint crucial proteins or hub nodes within complex systems. Consequently, it has become an invaluable tool for investigating the pharmacological profiles of polyherbal formulations and traditional therapeutic systems [14,15,16,17,18,19].

In continuation of our research project to unravel the mechanisms of action and targets of the bioactive compounds in polyherbal remedies used in TTM, we now describe our study of BLW for the treatment of TF, focusing on its anti-inflammatory actions. The integrated network pharmacology and molecular docking, in combination with pharmacological activity assays, particularly the inhibition of NO production in LPS-induced RAW 264.7 macrophages, were used for these purposes. The results obtained from this study can provide concrete scientific evidence to support the clinical use of this polyherbal remedy.

## 2. Results

### 2.1. Screening of Bioactive Compounds and BLW-Related Targets Based on ADME

An extensive literature search of the constituents of the five plants belonging to the BLW remedy, viz. *C. micracantha*, *C. indicum*, *F. racemosa*, *H. perforata*, and *T. triandra*, revealed that 32 compounds possess anti-inflammatory activity. However, only 15 compounds satisfied the requirements for drug-likeness and gastrointestinal (GI) absorption criteria, while the remaining 17 compounds with insufficient GI absorption (Supplementary Table S1) were not included. Following this selection, potential protein targets for the eligible compounds were predicted. After removal of redundant entries, a total of 495 unique targets associated with the active compounds were established. Notably, harrisolanol A was the sole compound that exhibited no target prediction probability. Comprehensive details are provided in Supplementary Table S2.

### 2.2. Obtention of Potential Therapeutic Targets of BLW for Treatment of TF

A total of 8139 disease-related targets were identified by employing “toxic fever” as a primary search descriptor across multiple genomic and pharmacological repositories. This comprehensive collection included 8031 targets retrieved from GeneCards, 11 from the Online Mendelian Inheritance in Man (OMIM) database, and 97 from PharmGKB. The complete list of these identified targets is detailed in Supplementary Table S3. To determine the pharmacological intersection, the targets associated with the BLW remedy were compared with those related to TF. This comparative analysis led to the identification of 88 overlapping targets, which were established as the candidate therapeutic target candidates for BLW in managing TF.

### 2.3. Protein–Protein Interaction (PPI) Construction and Cluster Analysis

**Figure 1:**
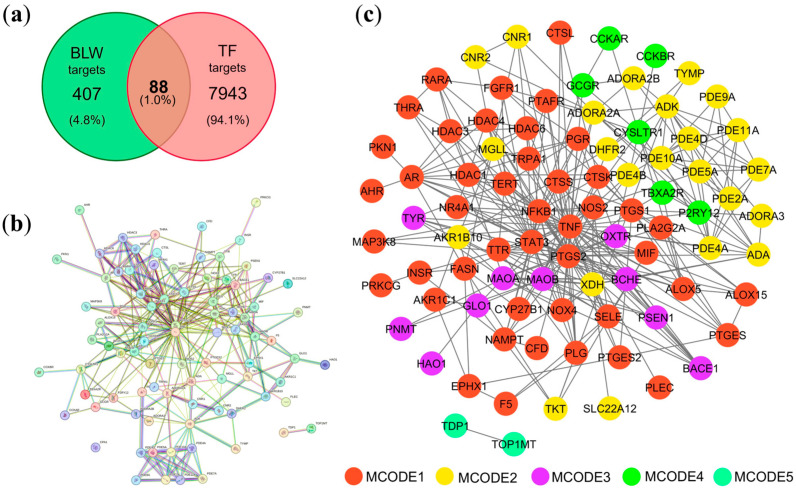
Network pharmacological analysis of overlapping genes between BLW and TF-related targets. (**a**) Venn diagram illustrating the intersection of targets. (**b**) Protein–protein interaction (PPI) network constructed using the STRING database. (**c**) Network topology analysis performed with Cytoscape. Circles of different colors represent distinct MCODE clusters.

**Figure 2:**
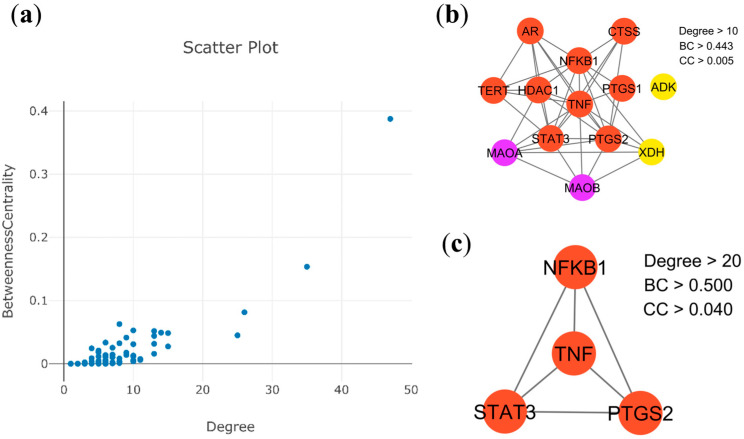
Systematic identification of hub genes within the PPI network. (**a**) Scatter plot illustrating the distribution of node degree values across the network. (**b**) Extraction of candidate genes possessing a degree threshold greater than 10. (**c**) Visualization of the four identified hub genes. The various colored circles indicate the specific MCODE clusters to which each protein belongs.

**Figure 3:**
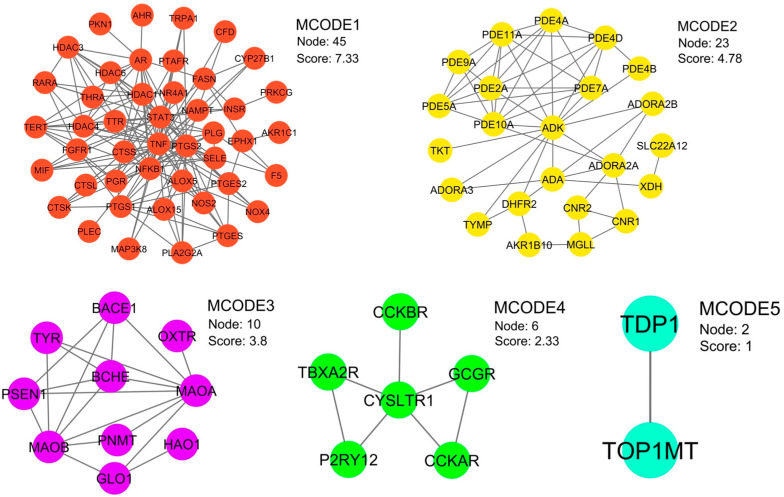
Functional categorization of MCODE sub-networks via gene ontology (GO) enrichment analysis. The visualization illustrates distinct protein clusters identified through modular analysis, with each MCODE network distinguished by a unique color assigned to its respective nodes and edges.

The interaction landscape was mapped among the 88 therapeutic target candidates through a PPI network, as shown in Figure 1c. This topological framework comprised 86 nodes, interconnected by 321 edges, and exhibited an average node degree of 7.74 and a local clustering coefficient of 0.245. By applying rigorous topological filtering as shown in Figure 2b,c, four central hub genes were prioritized, i.e., TNF (tumor necrosis factor-α), PTGS2 (prostaglandin-endoperoxide synthase 2), STAT3 (signal transducer and activator of transcription 3), and NFKB1 (nuclear factor kappa subunit 1) (Table 1). These hub proteins, which function as key cytokines and enzymes, are integral to the modulation of various biological activities, including signal transduction and oxidoreduction processes. Due to their exceptional network metrics, specifically their high degree, betweenness centrality, and clustering coefficients, these genes were identified as the primary core targets within the global network (Supplementary Table S4). Furthermore, module analysis using the Molecular Complex Detection (MCODE) algorithm revealed ten distinct functional clusters within the network (Figure 3, Table 2). Five of these MCODE-derived modules were further characterized by their unique biological roles. Specifically, MCODE1 exhibited a primary association with the arachidonic acid metabolism pathway, alongside involvement in ovarian steroidogenesis and the pathogenesis of African trypanosomiasis. In contrast, MCODE2 demonstrated significant correlations with purine and nucleotide metabolic processes, as well as pathways linked to morphine addiction. MCODE3 was linked to the metabolic pathways of amino acids, specifically phenylalanine, tyrosine, and histidine. Finally, MCODE4 was involved in the calcium signaling pathway and neuroactive ligand-receptor interactions, as detailed in Table 2.

### 2.4. GO and KEGG Pathway Enrichment Analysis

**Figure 4:**
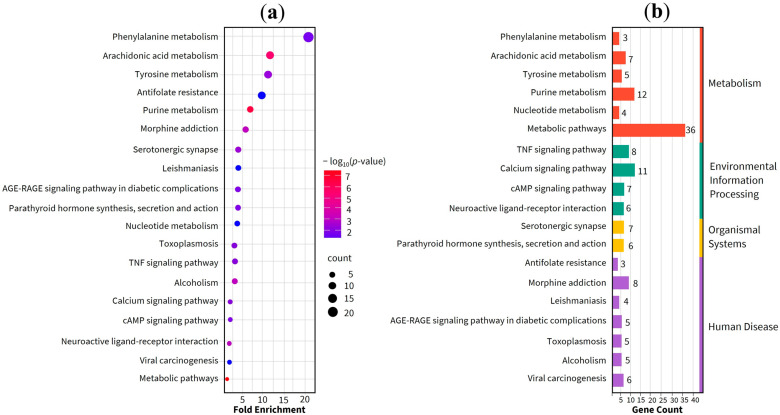
KEGG pathway enrichment analysis. (**a**) The top 30 enriched KEGG pathways based on enrichment score. (**b**) Classification of KEGG pathways and their corresponding gene counts.

**Figure 5:**
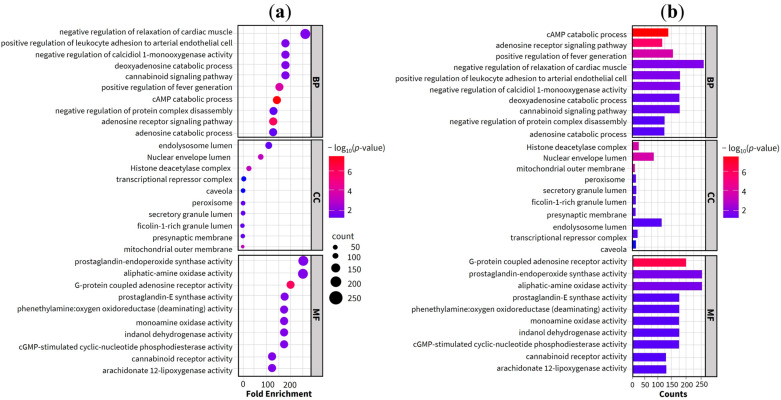
GO enrichment and signaling pathway analysis. (**a**) Bubble plot showing the top 10 enriched terms within the Biological Process (BP), Cellular Component (CC), and Molecular Function (MF) categories. (**b**) Bar plot illustrates the significantly enriched signaling pathways.

**Figure 6:**
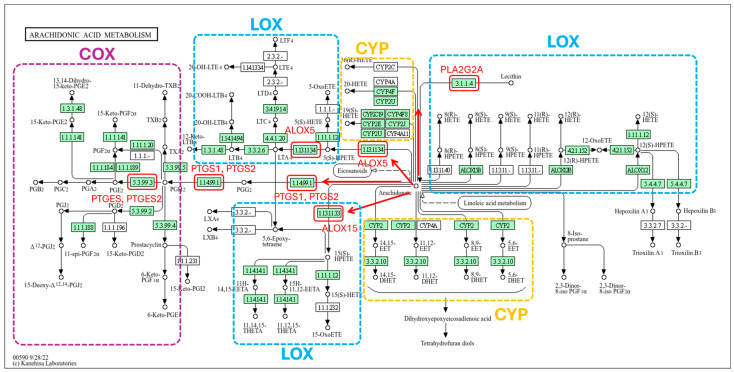
Arachidonic acid metabolism pathway. Red-highlighted boxes represent potential targets of BLW associated with TF. The red arrows indicate the gene pathways involved in the mechanism. Colored dashed lines demarcate the specific metabolic branches: violet for the cyclooxygenase (COX) pathway, blue for the lipoxygenase (LOX) pathway, and yellow for the cytochrome P450 (CYP) signaling pathway.

The enrichment analysis results revealed a total of 19 significant signaling pathways within the KEGG (Kyoto Encyclopedia of Genes and Genomes) database. Furthermore, the gene ontology (GO) enrichment results yielded a total of 194 significant terms, comprising 133 biological processes (BP), 23 molecular functions (MF), and 38 cellular components (CC). A comprehensive list and detailed description of these functional categories are provided in Supplementary Table S5. The signaling pathways are visualized in the bubble plot in Figure 4, while Figure 5 illustrates the top 10 GO terms across the BP, MF, and CC categories. In these plots, the bubble dimensions correspond to the count of enriched genes, and the color intensity reflects the *p*-values. Specifically, darker shades represent higher statistical significance with smaller *p*-values, and larger bubbles indicate a greater number of enriched therapeutic genes, suggesting a more robust association with the treatment of TF. According to the KEGG pathway enrichment findings, the most significantly enriched pathways included phenylalanine metabolism, arachidonic acid metabolism, and tyrosine metabolism. These pathways were prominently featured within the KEGG pathway network as shown in Figure 4. Additionally, Figure 6 highlights the arachidonic acid metabolism signaling pathway, specifically identifying potential targets involved in the condition.

In the BP category, the three most significant terms identified were the negative regulation of cardiac muscle relaxation, the negative regulation of calcidiol 1-monooxygenase activity, and the positive regulation of leukocyte adhesion to arterial endothelial cells. For the MF category, significant enrichment was observed in prostaglandin-endoperoxide synthase (PTGS) activity, aliphatic amine oxidase activity, and G-protein coupled adenosine receptor activity. Regarding the CC category, the endolysosome lumen, nuclear envelope lumen, and histone deacetylase complex emerged as the most enriched terms.

### 2.5. Investigation of the Possible Therapeutic Targets of BLW for TF Treatment

**Figure 7:**
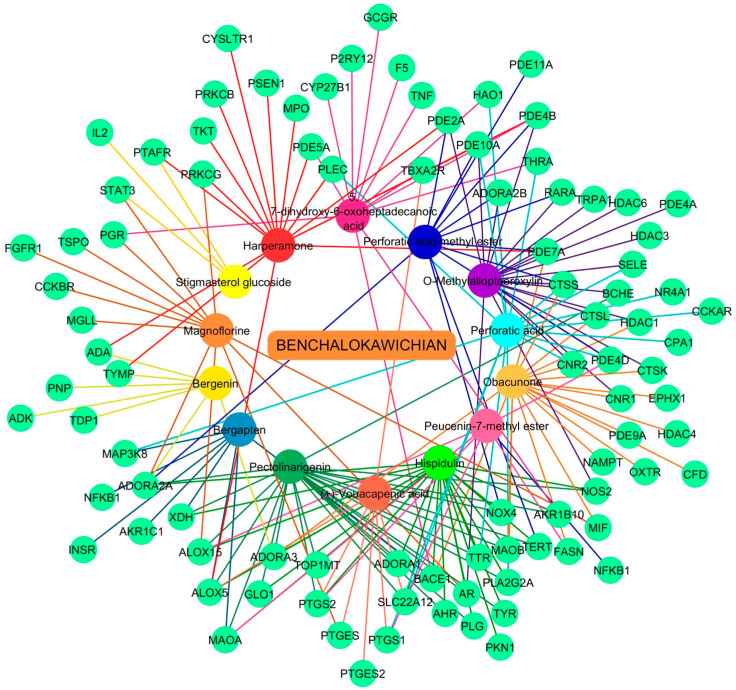
Network visualization illustrating the interactions between bioactive compounds and therapeutic targets for TF treatment. In this network, green circles indicate specific genes that serve as both TF-related markers and targets for the identified active constituents. Simultaneously, colored circles are utilized to distinguish the various bioactive compounds derived from the constituent medicinal plants.

The complex interactions between the identified bioactive constituents and their potential therapeutic targets are shown in Figure 7. This pharmacological network comprised 106 nodes and 189 edges, which collectively represent the binding associations between 14 active compounds and 88 unique targets. Upon analysis of the network topology, specific constituents, including hispidulin and pectolinarigenin of the BLW remedy exhibited the highest degree values (degree = 24).

### 2.6. Molecular Docking of Key Targets

**Figure 8:**
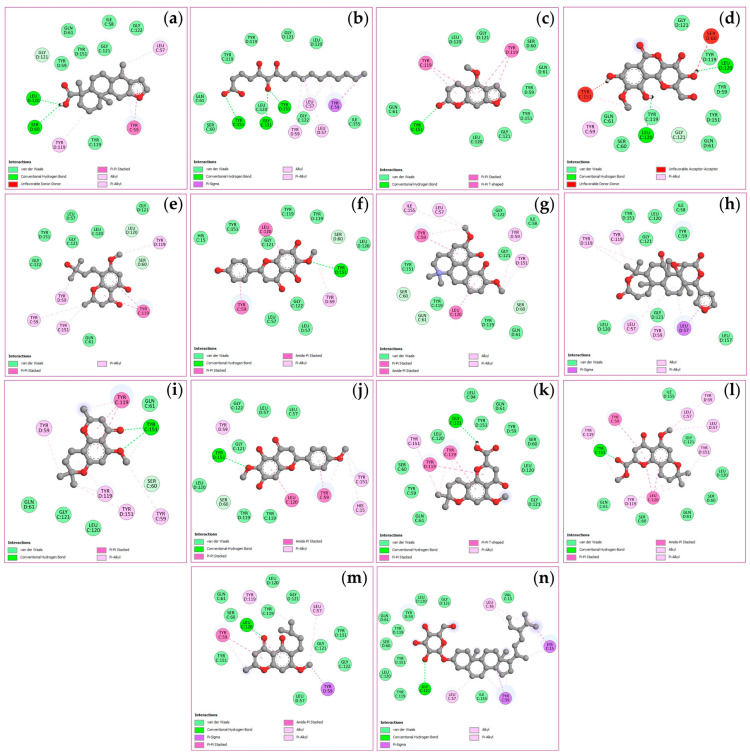
Visualization of molecular docking results for TNF. The molecular docking models depict the interactions between bioactive compounds and TNF: (+)-vouacapenic acid (**a**); 5,7-dihydroxy-6-oxoheptadecanoic acid (**b**); bergapten (**c**); bergenin (**d**); harperamone (**e**); hispidulin (**f**); magnoflorine (**g**); obacunone (**h**); *O*-methylalloptaeroxylin (**i**); pectolinarigenin (**j**); perforatic acid (**k**); perforatic acid methyl ester (**l**); peucenin-7-methyl ether (**m**); stigmasterol glucoside (**n**). Dash lines represent the bond interactions between bioactive compounds and proteins.

**Figure 9:**
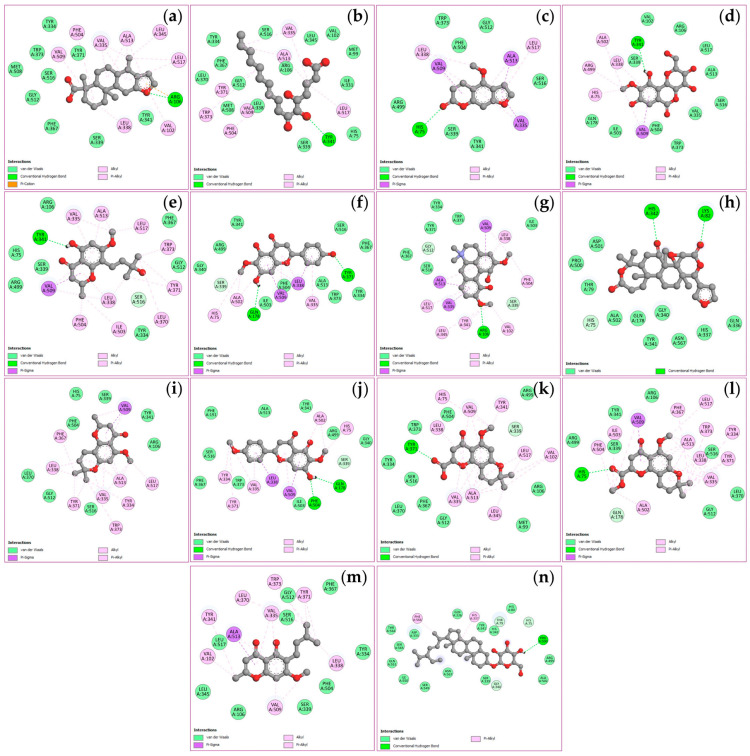
Visualization of molecular docking results for PTGS2. The molecular docking models depict the interactions between bioactive compounds and PTGS2: (+)-vouacapenic acid (**a**); 5,7-dihydroxy-6-oxoheptadecanoic acid (**b**); bergapten (**c**); bergenin (**d**); harperamone (**e**); hispidulin (**f**); magnoflorine (**g**); obacunone (**h**); *O*-methylalloptaeroxylin (**i**); pectolinarigenin (**j**); perforatic acid (**k**); perforatic acid methyl ester (**l**); peucenin-7-methyl ether (**m**); stigmasterol glucoside (**n**). Dash lines represent the bond interactions between bioactive compounds and proteins.

**Figure 10:**
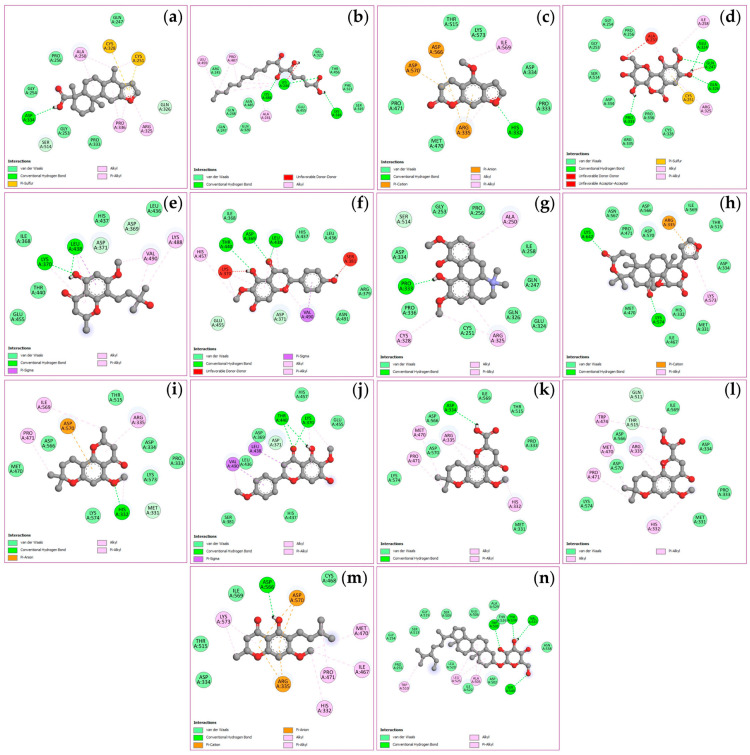
Visualization of molecular docking results for STAT3. The molecular docking models depict the interactions between bioactive compounds and STAT3: (+)-vouacapenic acid (**a**); 5,7-dihydroxy-6-oxoheptadecanoic acid (**b**); bergapten (**c**); bergenin (**d**); harperamone (**e**); hispidulin (**f**); magnoflorine (**g**); obacunone (**h**); *O*-methylalloptaeroxylin (**i**); pectolinarigenin (**j**); perforatic acid (**k**); perforatic acid methyl ester (**l**); peucenin-7-methyl ether (**m**); stigmasterol glucoside (**n**). Dash lines represent the bond interactions between bioactive compounds and proteins.

**Figure 11:**
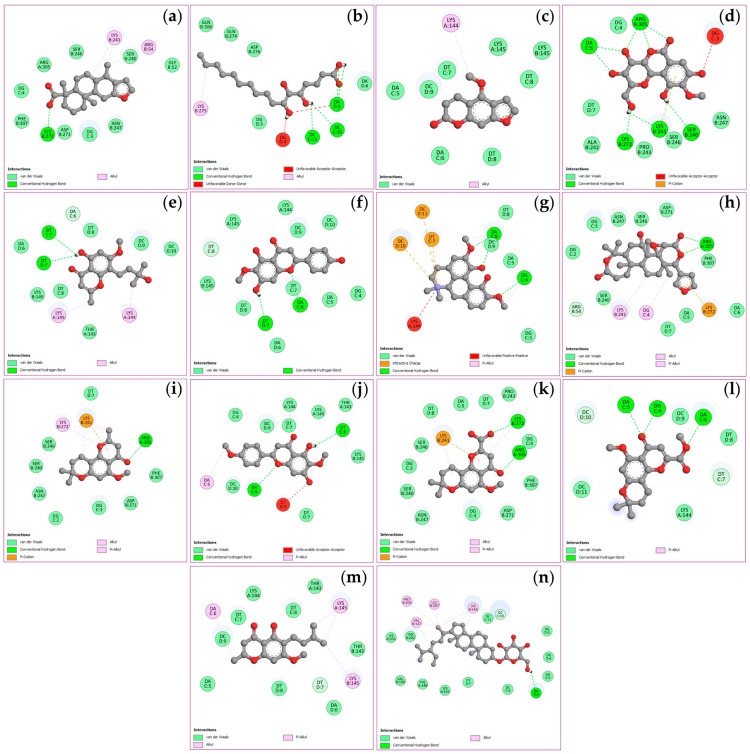
Visualization of molecular docking results for NFKB1. The molecular docking models depict the interactions between bioactive compounds and NFKB1: (+)-vouacapenic acid (**a**); 5,7-dihydroxy-6-oxoheptadecanoic acid (**b**); bergapten (**c**); bergenin (**d**); harperamone (**e**); hispidulin (**f**); magnoflorine (**g**); obacunone (**h**); *O*-methylalloptaeroxylin (**i**); pectolinarigenin (**j**); perforatic acid (**k**); perforatic acid methyl ester (**l**); peucenin-7-methyl ether (**m**); stigmasterol glucoside (**n**). Dash lines represent the bond interactions between bioactive compounds and proteins.

The docking simulations evaluated binding affinities for four key targets, viz. TNF, PTGS2, STAT3, and NFKB1, with binding energies spanning from −4.5 to −11.1 kcal/mol as shown in Table 3. Analysis of the TNF receptor revealed that obacunone possessed the strongest binding affinity at −9.1 kcal/mol, followed by (+)-vouacapenic acid and stigmasterol glucoside, both at −8.9 kcal/mol, and magnoflorine at −7.9 kcal/mol. In the case of the PTGS2 receptor, hispidulin demonstrated the optimal docking score of −9.4 kcal/mol, followed by perforatic acid methyl ester at −9.3 kcal/mol, while both *O*-methylalloptaeroxylin and perforatic acid recorded values of −8.9 kcal/mol. For the STAT3 receptor, obacunone exhibited the highest affinity at −9.3 kcal/mol, followed by hispidulin and stigmasterol glucoside at −7.7 kcal/mol, and (+)-vouacapenic acid at −7.5 kcal/mol. Furthermore, obacunone showed the most potent interaction with the NFKB1 receptor at −11.1 kcal/mol, surpassing pectolinarigenin at −9.6 kcal/mol, magnoflorine at −9.5 kcal/mol, and hispidulin at −9.3 kcal/mol. Due to the unavailability of the 3D structure for harrisolanol A in the PubChem database, it was excluded from this analysis. Figure 8, Figure 9, Figure 10 and Figure 11 show the molecular interactions and specific bonding patterns between these active constituents or reference drugs and their target proteins. The characterization of hydrogen bond interactions can be represented through various structural formats, including line, ball-and-stick, and cycle models, to enhance the observation of molecular binding sites. In this study, the specific hydrogen bonding patterns between the target proteins and the ligands are visualized using green dashed line representations. Detailed interaction data and bonding parameters for each protein-ligand complex are shown in Figure 8, Figure 9, Figure 10 and Figure 11.

### 2.7. Characterization of the BLW Extract

**Figure 12:**
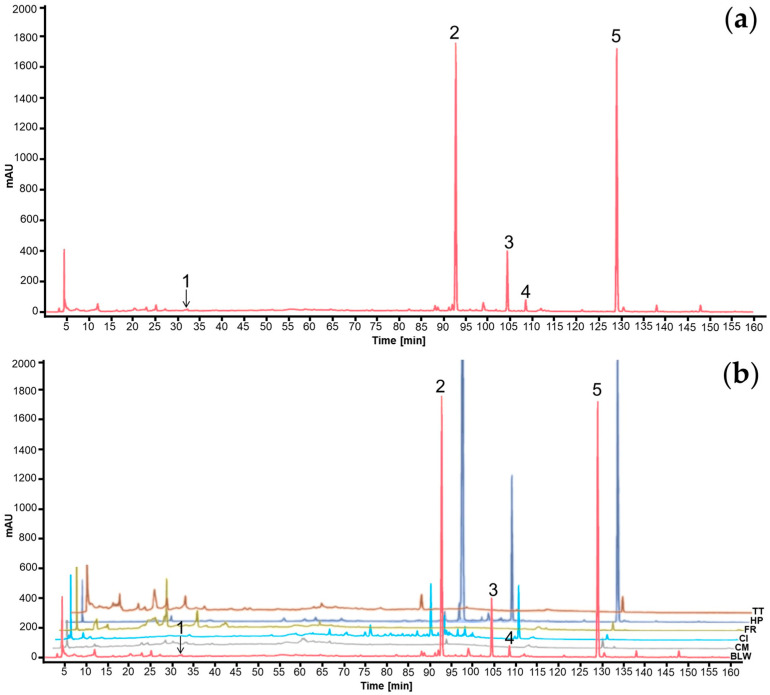
HPLC chromatogram of biomarker compounds for BLW extract (**a**) exhibiting the peaks of bergenin (**1**), perforatic acid (**2**), *O*-methylalloptaeroxylin (**3**), pectolinarigenin (**4**), and peucenin-7-methyl ether (**5**). HPLC chromatograms of Benjalokawichian (BLW) and its component plants (**b**), *Capparis micracantha* (CM), *Clerodendrum indicum* (CI), *Ficus racemosa* (FR), *Harrisonia perforata* (HP), *Tiliacora triandra* (TT).

**Figure 13:**
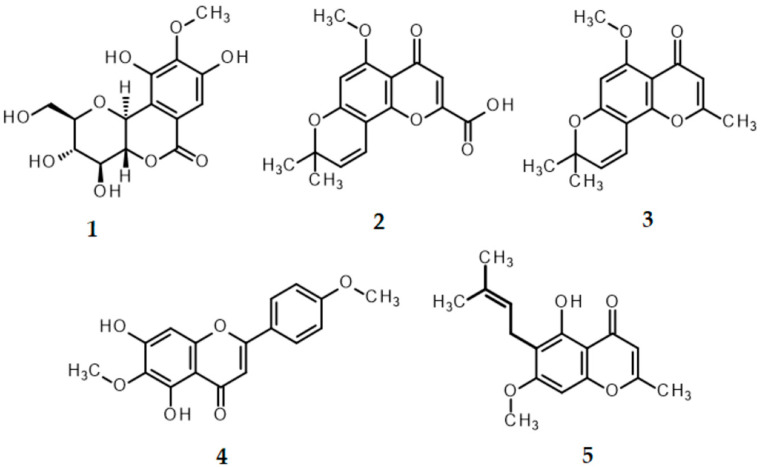
Chemical structures of the markers for the BLW extract: bergenin (**1**), perforatic acid (**2**), *O*-methylalloptaeroxylin (**3**), pectolinarigenin (**4**), and peucenin-7-methyl ether (**5**).

The HPLC chromatograms showing the major biomarker compounds are presented in Figure 12. The specific retention times for the five identified markers were determined as follows: bergenin (**1**) at 32.4 min, perforatic acid (**2**) at 92.9 min, *O*-methylalloptaeroxylin (**3**) at 104.6 min, pectolinarigenin (**4**) at 108.7 min, and peucenin-7-methyl ether (**5**) at 129.3 min. The chemical structures of these BLW biomarker compounds are depicted in Figure 13. Among these markers, perforatic acid (**2**), *O*-methylalloptaeroxylin (**3**), and peucenin-7-methyl ether (**5**) represent the primary constituents of *H. perforata*, while bergenin (**1**) and pectolinarigenin (**4**) are the major characteristic compounds of *F. racemosa* and *C. indicum*, respectively. In this study, obacunone was not detected in the BLW extract; the corresponding HPLC chromatogram is provided in Supplementary Figure S1.

The amount of the major compounds in the BLW extract was determined using a calibration curve of the reference standard. The linearity of the developed method was determined by *R*^2^, which was assessed in six concentration levels as follows. Bergenin showed a linear equation of y = 31.112x + 177.07 with the *R*^2^ value of 1.0000. Perforatic acid showed y = 33.193x − 224.22 with the *R*^2^ value of 0.9999. *O*-methylalloptaeroxylin showed y = 9.7555x + 173.69, with the *R*^2^ value of 0.9996. Pectolinarigenin showed y = 41.603x − 5.8451 and the *R*^2^ value of 1.0000. Peucenin-7-methyl ether showed y = 75.721x + 598.97 with the *R*^2^ value of 0.9997. The BLW extract contained the highest amount of perforatic acid at 80.89 mg/g extract, followed by *O*-methylalloptaeroxylin and peucenin-7-methyl ether. On the contrary, pectolinarigenin, and bergenin were present in much lower amounts in the BLW extract (Table 4).

### 2.8. Effects of the BLW Extract and Biomarker Compounds on NO Production in LPS-Induced RAW 264.7 Macrophages

**Figure 14:**
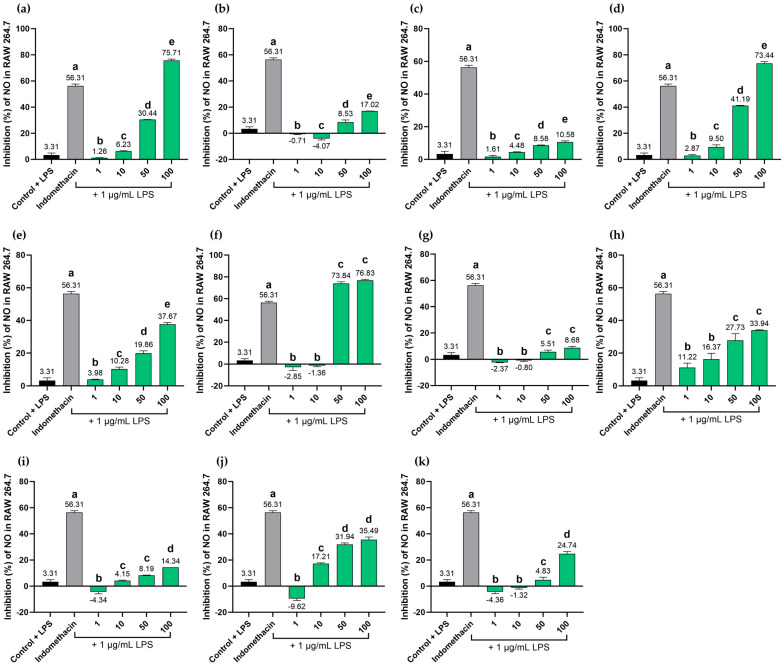
Comparative inhibitory effects of BLW extract, individual plant extracts, and marker compounds on NO production. The evaluation was performed using LPS-stimulated RAW 264.7 macrophages at concentration ranges from 1 to 100 μg/mL for the following samples: BLW extract (**a**); *C. micracantha* extract (**b**); *C. indicum* extract (**c**); *F. racemosa* extract (**d**); *H. perforata* extract (**e**); *T. triandra* extract (**f**); bergenin (**g**); perforatic acid (**h**); *O*-methylalloptaeroxylin (**i**); pectolinarigenin (**j**); peucenin-7-methyl ether (**k**). Bars labeled with different letters represent statistically significant differences (*p* < 0.01). Data are presented as mean ± SD (n = 3). Indomethacin (100 μg/mL) served as the positive control.

The BLW extract effectively suppressed nitric oxide (NO) production in LPS-stimulated RAW 264.7 macrophages, yielding an IC_50_ value of 69.10 µg/mL. Notably, at a concentration of 100 µg/mL, the BLW extract demonstrated superior potency compared to the positive control, indomethacin, which had an IC_50_ of 73.42 µg/mL. The inhibitory activity of the BLW extract was dose-dependent within the concentration range of 50 to 100 µg/mL. Among the individual components of BLW, the extract of *T. triandra* exhibited the most significant inhibitory effect, with an IC_50_ of 45.71 µg/mL, followed by the extract of *F. racemosa*, with an IC_50_ of 65.71 µg/mL. Conversely, the remaining single-herb extracts showed limited activity, with IC_50_ values exceeding 100 µg/mL. It is worth noting that the *H. perforata* extract displayed a dose-dependent response in the concentration range of 10 to 100 µg/mL. Interestingly, all tested marker compounds were significantly less effective than indomethacin when evaluated at 100 µg/mL. However, specific compounds showed characteristic dose-dependent trends as shown in Figure 14. Perforatic acid exhibited dose-dependent inhibition at concentrations of 1 to 100 µg/mL, while peucenin-7-methyl ether exhibited a similar behavior at concentrations of 10 to 100 µg/mL.

### 2.9. Evaluation of Cytotoxicity in RAW 264.7 Macrophages of the BLW Extract and Biomarker Compounds

**Figure 15:**
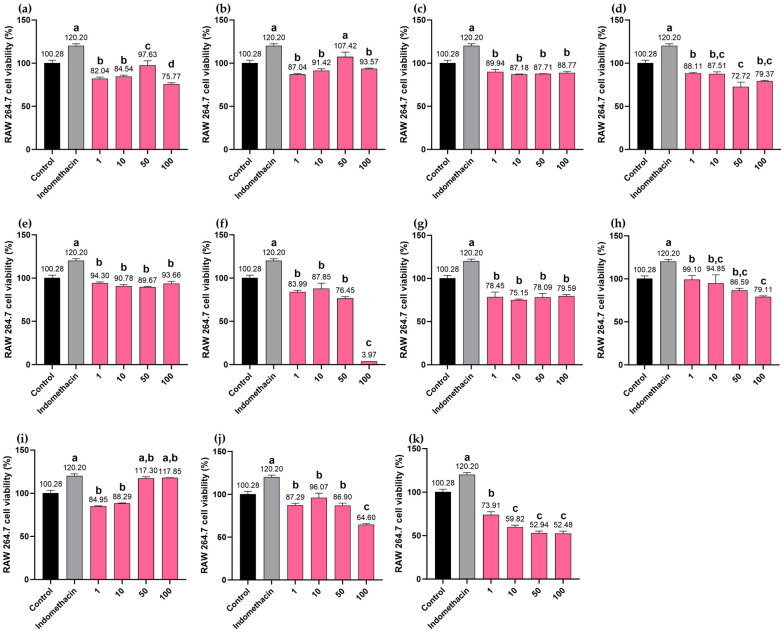
Assessment of cell viability in RAW 264.7 macrophages following treatment with BLW extract and marker compounds. Macrophages were incubated with varying concentrations (1 to 100 μg/mL) of the following: BLW extract (**a**); *C. micracantha* extract (**b**); *C. indicum* extract (**c**); *F. racemosa* extract (**d**); *H. perforata* extract (**e**); *T. triandra* extract (**f**); bergenin (**g**); perforatic acid (**h**); *O*-methylalloptaeroxylin (**i**); pectolinarigenin (**j**); peucenin-7-methyl ether (**k**). Bars marked with different letters denote statistically significant differences (*p* < 0.01). Results are presented as mean ± SD (n = 3). Indomethacin (100 μg/mL) was employed as the positive control.

The cytotoxicity of the BLW extract and the extracts of its individual components was evaluated using RAW 264.7 macrophages. Results indicated that the BLW extract, along with extracts of *C. micracantha*, *C. indicum*, *F. racemosa*, and *H. perforata*, as well as the biomarkers, bergenin, perforatic acid, and *O*-methylalloptaeroxylin, did not affect cell viability within the concentration range of 1 to 100 µg/mL. However, at the maximum tested concentration of 100 µg/mL, several treatments, viz., the BLW extract, bergenin, perforatic acid, pectolinarigenin, and peucenin-7-methyl ether, resulted in significantly lower cell viability compared to indomethacin (a positive control), except for *O*-methylalloptaeroxylin, which maintained a percentage of cell viability comparable to that of indomethacin. Notably, the extract of *T. triandra* exhibited cytotoxic effects when administered at 100 µg/mL. Furthermore, the cytotoxicity of pectolinarigenin exhibited a clear dose-dependent trend, where cell viability decreased as the concentration increased, as shown in Figure 15.

## 3. Discussion

Toxic fever (TF) is characterized by a sudden and severe high fever, which is frequently associated with serious infections and inflammatory processes [10]. In Thai traditional medicine, a polyherbal remedy known as BLW has been used to alleviate TF. This remedy is also reported to modulate inflammatory conditions [11,12]. Polyherbal remedies are gaining popularity in both modern and traditional medicine. The underlying logic of these botanical formulations is centered on the interaction of diverse bioactive constituents. This multicomponent strategy is designed to improve therapeutic efficacy by employing a holistic approach to health and wellness. The synergistic interaction among the constituents of different herbs can complement and potentiate individual therapeutic effects, minimize adverse reactions, and improve overall treatment efficacy. Polyherbal remedies provide multi-targeted treatment, addressing the root causes like oxidative stress or immune system dysregulation [6,20,21]. Despite their advantages, many aspects, such as the mechanism of action, identification of active compounds, standardization, quality control, and regulatory approval, remain serious obstacles to the acceptance of polyherbal remedies in mainstream medicine [20]. Network pharmacology offers a systematic framework for interpreting the complex interactions among bioactive compounds, molecular targets, and diseases. It facilitates the elucidation of multi-target mechanisms underlying many traditional medicines, particularly Traditional Chinese Medicine (TCM), thereby revealing potential therapeutic pathways from a holistic and integrative perspective [22].

This research applied an integrated approach, combining network pharmacology and molecular docking to investigate the potential mechanisms by which the BLW remedy exerts its effects in the treatment of TF. Through a systematic process of screening bioactive constituents, identifying therapeutic protein targets, and evaluating protein–protein interaction networks, our study allowed us to map the complex interplay between the remedy and the disease.

Widely reported metabolic changes during fever, metabolomics, and network pharmacology analyses have revealed distinct biomarkers and gene targets under varying conditions [14,15,16]. Literature review suggests that BLW comprises 15 bioactive constituents with 495 genes. This study identified 8031 TF-associated genes, 88 of which overlapped with BLW targets. These common genes regulate vital processes such as inflammation, apoptosis, and cell differentiation. Notably, TNF, PTGS2, STAT3, and NFKB1 were identified as the highest-connectivity targets within the network. Their strong binding affinities with the 17 BLW compounds were further validated through molecular docking, confirming their central role in the therapeutic mechanism.

Network pharmacology and molecular docking are established methods for identifying bioactive constituents and their corresponding targets in studies of fever. A prominent example includes the analysis of Xiaochaihu Decoction, where 726 bioactive compounds were retrieved from the traditional Chinese medicine systems pharmacology (TCMSP) database. This remedy is linked to 677 molecular targets. Subsequent research identified 7305 fever-associated genes, eventually narrowing them down to 400 core targets with direct therapeutic relevance to febrile conditions. Previous research underscores the efficacy of network pharmacology in identifying bioactive compounds for febrile conditions. For instance, GAPDH, AKT1, INS, IL6, and VEGFA were established as primary targets in related studies [14]. Similarly, analysis of Bai Hu Tang was able to identify 120 active compounds and 2176 targets, leading to the selection of 34 therapeutic candidates, including TNF, CASP1, PTGS2, and IL6R [15]. Furthermore, Zi Xue Powder was found to contain 126 active compounds linked to 707 potential targets, with ALB, AKT1, INS, TNF, and IL6 emerging as pivotal proteins [16]. Despite differences in herbal composition and specific chemical constituents, these remedies share common biological mechanisms. Previous studies consistently highlighted the regulation of cell proliferation, apoptosis, and inflammation as central to the antipyretic effect [14,15,16]. Notably, while the current study identified a more focused set of active compounds, it successfully mapped a significantly larger number of target genes than previous reports. Most importantly, this research reveals that the therapeutic potential of BLW is highly enriched in the phenylalanine and arachidonic acid metabolism pathways, both of which play critical roles in modulating fever and inflammatory responses.

The 88 identified therapeutic targets form a complex and interconnected network where nodes and edges represent individual proteins and their interactions. Within this system, higher node degrees indicate more significant functional roles. Notably, four core proteins consisting of TNF, PTGS2, STAT3, and NFKB1 serve as central hubs. These proteins play synergistic roles in regulating infection, inflammation, cell proliferation, and apoptosis in TF. Their clustering into the primary functional module underscores their importance as pivotal therapeutic targets for the BLW remedy. Current evidence indicates that fever is primarily initiated by pro-inflammatory cytokines, including TNF, IL6, and IL1B. These mediators trigger the activation of phospholipase A2 (PLA_2_), which facilitates the conversion of phospholipids into arachidonic acid. This precursor is subsequently transformed into PGH_2_ via the enzymatic action of COX-2. Furthermore, the elevated expression of COX-2 and mPGES-1 promotes the synthesis of PGE_2_ within the central nervous system, which acts as a key thermal regulator during the febrile response. As a principal mediator of fever, PGE_2_ binds to EP3 receptors on hypothalamic neurons, signaling the brain to elevate the body’s thermal set point [1,2,4,5]. TNF contributes to fever induction through mechanisms dependent on IL6 plasma levels but independent of IL1B. While exogenous TNF-induced fever involves peripheral IL1B release, intravenous injection of recombinant human TNF (rhTNF) triggers pyrogenicity through glutathione-associated PGE_2_ production. Beyond the direct inflammatory response, IL6 interacts with endothelial receptors in the brain to trigger COX-2 expression, a process primarily mediated by the STAT3 signaling pathway. Concurrently, IL1B promotes COX-2 production within cerebral endothelial cells through the activation of the NF-κB (nuclear factor kappa-light-chain-enhancer of activated B cells) pathway. The convergence of these intracellular signals significantly enhances PGE_2_ synthesis, thereby amplifying the febrile response [23].

On the basis of ADME (absorption, distribution, metabolism, and excretion) criteria with parameters set for high gastrointestinal absorption and a drug-likeness (DL) of 1, several plant constituents in BLW are considered highly promising. Specifically, 5,7-dihydroxy-6-oxoheptadecanoic acid, harperamone, hispidulin, magnoflorine, obacunone, *O*-methylalloptaeroxylin, pectolinarigenin, perforatic acid, and perforatic acid methyl ester were found to interact with ten or more targets. These findings suggest that these compounds may contribute significantly to the pharmacological activity of BLW in treating TF. It is essential to note that the therapeutic efficacy of BLW stems from the synergistic action of its complex chemical composition rather than isolated constituents. Recent analysis identified perforatic acid and *O*-methylalloptaeroxylin as major components, while pectolinarigenin was classified as a minor constituent. Although present in relatively lower concentrations, *O*-methylalloptaeroxylin demonstrated robust anti-inflammatory properties. This was evidenced by its potent inhibition of NO secretion in LPS-stimulated RAW 264.7 cells, with an IC_50_ of 7.92 µg/mL [24]. Furthermore, pectolinarigenin has been shown to modulate the inflammatory response by inhibiting LPS-induced NF-κB activation through the stabilization of IκB-α (nuclear factor of kappa light polypeptide gene enhancer in B-cells inhibitor alpha). This compound regulates the NF-κB/Nrf2 signaling pathways, subsequently suppressing the synthesis of pro-inflammatory mediators, viz., iNOS, COX-2, IL6, IL1B, and TNF [25]. These observations are further reinforced by prior investigations into pectolinarigenin, a key constituent isolated from BLW. This compound has been documented to possess robust inhibitory effects on NO secretion, exhibiting a potent IC_50_ value of 7.15 µg/mL [24].

GO enrichment analysis was conducted to characterize the functional roles and molecular mechanisms of potential therapeutic targets, specifically focusing on their involvement in overcoming TF resistance. The BP associated with BLW targets were primarily linked to cardiac function, apoptosis regulation, cell proliferation, and the inflammatory response. These findings are particularly relevant to TF, which is characterized by high fever often resulting from severe infection and systemic inflammation [10]. Key targets identified in this study, including cytokines like TNF and inflammatory enzymes such as PTGS2, play critical roles in modulating the inflammatory microenvironment of TF [1,2,4,5]. Regarding MF, the targets exhibited specific activities such as prostaglandin-endoperoxide synthase, aliphatic-amine oxidase, and G-protein coupled adenosine receptor activity. These functions also encompassed broader categories, viz., protein, enzyme, and energy substance binding. Furthermore, CC analysis localized these activities to specific substructures like the endolysosome lumen, nuclear envelope lumen, and histone deacetylase complex. These intracellular sites are intrinsically associated with inflammatory pathways, cellular turnover, pyretic responses, and RNA transcription. Such localization supports the therapeutic potential of BLW in managing TF by modulating specific proteins involved in its pathophysiology, notably TNF, PTGS2, STAT3, and NFKB1 [1]. These results reinforce the multi-dimensional approach of the BLW remedy in targeting the molecular foundations of fever and inflammation.

KEGG pathway enrichment analysis revealed that the anti-TF properties of the BLW are primarily mediated through specific metabolic signaling. Critical gene targets were significantly localized within the arachidonic acid, phenylalanine, and tyrosine metabolism pathways, suggesting a multi-targeted biochemical mechanism for its therapeutic efficacy. Modulating these pathways directly influences biological processes associated with infection, inflammation, and cellular turnover, suggesting that BLW may also play a role in mitigating chemotherapy resistance. The phenylalanine and tyrosine metabolism pathways are well-recognized for their involvement in inflammatory diseases and severe fever [26]. Specifically, inflammatory states frequently lead to a marked increase in the serum phenylalanine-to-tyrosine ratio, a phenomenon observed in various infectious diseases, *viz*., viral encephalitis and yellow fever [27]. Furthermore, the arachidonic acid metabolism pathway serves as a theoretical bridge, linking traditional “heat” theories with modern biochemical understanding of fever and inflammatory responses [1,2,3,4,5,6]. The therapeutic potential of BLW through these metabolic routes is supported by multiple studies. The ethanolic extract of BLW has been shown to suppress COX-2 expression and reduce PGE_2_ levels in IL1B-stimulated human umbilical vein endothelial cells [3]. Additionally, BLW effectively decreased TNF and IL1B levels in LPS-induced macrophages, with IC_50_ values of 103.96 and 60.09 µg/mL for TNF and PGE_2_ inhibition, respectively [28]. In vivo evidence further validated these findings. Oral administration of BLW powder at doses ranging from 300 to 3000 mg/kg reduced plasma TNF and IL1B in rats with systemic inflammation [29]. Regarding antipyretic efficacy, BLW powder was effective against yeast-induced fever [30]. Moreover, oral administration of BLW extract at doses of 300 mg/kg or within a range of 25 to 400 mg/kg exhibited potency comparable to acetylsalicylic acid in reducing rectum temperature in LPS-induced fever models [31].

Molecular docking revealed the interaction between compounds and targets in the network pharmacology. Results indicated that obacunone exhibited the strongest binding affinity with the TNF, STAT3, and NFKB1 receptors, which is consistent with the finding of Luo et al., who reported the suppression of the protein levels of pro-inflammatory cytokines (TNF, iNOS, COX-2) and phosphorylation of the NF-κB complex by obacunone [32]. Moreover, obacunone, isolated from the roots of *H. perforata*, was found to inhibit LPS-induced NO production in RAW 264.7 macrophages, with an IC_50_ of 83.61 µM [33], while hispidulin, a constituent of *C. indicum*, exhibited the strongest binding affinity with the PTGS2 receptor [34]. Yu et al. reported a significant reduction in the levels of NO, ROS, iNOS, COX-2, TNF, IL1B, IL6, and PGE_2_ by hispidulin, in a dose-dependent manner, suggesting this compound may inhibit neuroinflammatory responses through NF-κB pathway inhibition [35]. Additionally, key biomarkers within the BLW, specifically bergenin, perforatic acid, *O*-methylalloptaeroxylin, pectolinarigenin, and peucenin-7-methyl ether, demonstrated high binding affinities toward their respective protein targets.

In this work, the biomarkers used for HPLC analysis of the BLW extract are bergenin, perforatic acid, *O*-methylalloptaeroxylin, pectolinarigenin, and peucenin-7-methyl ether. However, obacunone was not detected. Bergenin, an isocoumarin glycoside, is a constituent of *F. racemosa* root [36,37], while the chromones perforatic acid, *O*-methylalloptaeroxylin, and peucenin-7-methyl ether are constituents of *H. perforata* root [38,39], and the isoflavone pectolinarigenin is a constituent of *C. indicum* root [40,41]. It is worth mentioning that all the biomarkers used to characterize the BLW extract in this study possess anti-inflammatory activity [24,33,42,43,44]. The HPLC chromatogram showed that perforatic acid, *O*-methylalloptaeroxylin, and peucenin-7-methyl ether are major peaks, while bergenin and pectolinarigenin constitute minor comounds. It is worth mentioning that some authors have used different markers such as pectolinarigenin [40,41], tiliacorinine, yanangcorinine [45], and lupeol [46,47] for HPLC fingerprint analysis of this polyherbal remedy. Although obacunone was not detected in our current study, its high binding affinity in the docking study remains theoretically significant, suggesting it could be a potent minor constituent or a ‘trace’ compound that contributes to the synergy.

Inflammation is one of the mechanisms of fever. In fever reactions, pro-inflammatory cytokines such as COX-2, TNF, IL1B, IL6, and NF-κB trigger the expression of the inducible nitric oxide synthase (iNOS) in monocytes or macrophages, neutrophil granulocytes, and many other cells. However, in the case of bacterial infection, endotoxin is another strong inducer of iNOS expression. Consequently, large amounts of NO are synthesized, exceeding the physiological NO production by up to 1000-fold [48,49,50,51]. The use of the LPS-induced RAW 264.7 macrophage model in this study serves as a critical proxy for understanding the early inflammatory phase of TF. In TTM, TF is characterized by systemic ‘heat’ and inflammation, which biochemically correlates with the overproduction of pro-inflammatory mediators. Macrophages are primary sources of NO and cytokines during an inflammatory response; thus, the inhibition of NO in this model reflects the ability of BLW to modulate the antipyretic stage of inflammation by downregulating the iNOS pathway [50]. In this study, the BLW extract potently inhibited NO production in LPS-induced RAW 264.7 macrophages, with an IC_50_ value of 69.10 µg/mL. This finding was in agreement with the studies by Juckmeta and Itharat (2012), who reported the IC_50_ values of 40.36 µg/mL [52], and by Kaewnoi et al. (2024), who reported the IC_50_ of 97.38 µg/mL for ethanol extracts of BLW in the same model [28]. *T. triandra* and *F. racemosa* were the only single-herb extracts to exhibit potent inhibition of NO production, with IC_50_ values of 45.71 and 65.71 µg/mL, respectively, while the remaining single-herb extracts showed only weak inhibitory activity. Our findings are also in agreement with the previous report by Juckmeta and Itharat, which used the ethanolic extracts of the same plants and the same assay model. They obtained the IC_50_ values for NO inhibition of 61.35 µg/mL for *C. micracantha*, 46.55 µg/mL for *C. indicum*, 53.16 µg/mL for *H. perforata*, and 54.65 µg/mL for *T. triandra*. Furthermore, they have also found that *F. racemosa* showed only weak inhibitory activity [52]. In this study, the individual biomarkers bergenin, perforatic acid, *O*-methylalloptaeroxylin, pectolinarigenin, and peucenin-7-methyl ether generally showed weak NO inhibitory activity (IC_50_ > 100 µg/mL). However, perforatic acid exhibited a dose-dependent inhibition of NO production at the concentration range of 1–100 µg/mL, while peucenin-7-methyl ether showed significant inhibitory effects within the 10–100 µg/mL concentration range.

The anti-inflammatory efficacy of bergenin, *O*-methylalloptaeroxylin, pectolinarigenin, and peucenin-7-methyl ether is well-documented in previous studies. Gao et al., in their study of the effects and mechanism of bergenin on the mammary glands during LPS-induced mastitis in a mouse model, have found that bergenin reduced the expression of NO, TNF, IL1B, and IL6 proinflammatory cytokines by inhibiting the activation of the NF-κB and MAPKs signaling pathways [42]. Intriguingly, although perforatic acid, *O*-methyllaloptaeroxyrin, and peucenin-7-methyl ether are major compounds that exhibited strong binding to proteins, they showed weak NO inhibitory activity. On the contrary, Juckmeta has found the inhibition of NO production in LPS-induced RAW 264.7 macrophages by *O*-methylalloptaeroxylin and pectolinarigenin, with IC_50_ values of 7.92 and 7.15 µg/mL, respectively [24], while Choodej et al. reported the NO inhibition by peucen-in-7-methyl ether, with an IC_50_ of 56.36 µM [33].

The extracts of BLW, *C. micracantha*, *C. indicum*, *H. perforata*, *F. racemosa* and the biomarkers, *viz.* bergenin, perforatic acid, and *O*-methylalloptaeroxylin showed no cytotoxicity against RAW 264.7 macrophages at concentrations where cell viability remained above 70%. However, pectolinarigenin and peucenin-7-methyl ether exhibited increased cytotoxicity at higher concentrations. Only the *T. triandra* extract displayed cytotoxicity at the highest tested concentration (100 µg/mL); however, the extract showed no cytotoxicity at a concentration of 50 µg/mL. These findings are consistent with previous reports of non-toxicity of the BLW extract in macrophage cell lines [28,52], in rats [29], and in humans [53].

The experimental results demonstrate a notable discrepancy between the potent anti-inflammatory activity of the BLW extract and the relatively weak NO inhibitory effects observed in its individual biomarkers. This phenomenon suggests that the therapeutic efficacy of BLW is not attributable to a single bioactive compound but rather arises from synergistic or additive interactions among its complex phytochemical constituents [54]. According to the principles of network pharmacology, herbal formulas exert their effects through a multi-compound and multi-target mechanism. This approach allows multiple low-affinity constituents to simultaneously modulate various nodes within a biological network, thereby achieving a robust overall effect that exceeds the sum of its individual parts [19,55]. In the context of BLW, while individual biomarkers showed limited direct inhibition of NO production, they may work in concert to suppress the inflammatory response by targeting complementary proteins such as TNF, COX-2, STAT3, NFKB1, and various pro-inflammatory cytokines [24,33,42]. This collective action highlights the importance of the ‘whole-extract’ approach in TTM, where the combination of ingredients serves to enhance efficacy and potentially reduce the concentration-dependent toxicity of any single component [24,56].

Despite the significant insights provided by the integration of network pharmacology and in vitro assays, this study has certain limitations. The RAW 264.7 cell model, while effective for screening NO inhibition, only partially represents the intricate ‘TF’ microenvironment characterized by systemic cytokine storms and hypothalamic thermoregulatory shifts. Consequently, future in vivo investigations using pyrogen-induced fever models (e.g., LPS or yeast-induced models) are essential to confirm the systemic antipyretic efficacy and to validate the predicted metabolic pathways within a complex biological system. Such studies will provide a more comprehensive understanding of how BLW modulates the thermoregulatory center to alleviate fever, further bridging the gap between traditional Thai medicine and evidence-based clinical application.

## 4. Materials and Methods

### 4.1. Screening for Potential Bioactive Compounds and BLW-Related Targets

To identify potential bioactive compounds in the BLW remedy, an extensive literature review was conducted using several electronic platforms, including PubMed/Medline, ScienceDirect, ISI Web of Science, and ClinicalTrials.gov. Moreover, the search for compounds from the five medicinal plants in BLW with reported anti-inflammatory or antipyretic properties from Thai research databases was also carried out. The identified constituents were screened via the SwissADME database (http://www.swissadme.ch/, accessed on 24 November 2023) [57], focusing on high gastrointestinal (GI) absorption and a drug-likeness score of 1. These criteria ensured the selection of molecules with optimal oral bioavailability. Chemical structures of the filtered compounds were subsequently retrieved from PubChem (https://pubchem.ncbi.nlm.nih.gov/, accessed on 24 November 2023) [58] in SMILES format. To identify potential therapeutic targets of the BLW formula, the SwissTargetPrediction database (http://www.swisstargetprediction.ch/, accessed on 24 November 2023) [59] was utilized. Only targets demonstrating a probability score greater than 0 were retained for further analysis.

### 4.2. Identification of TF-Related Targets

To identify genes associated with TF, data were integrated from three primary repositories: GeneCards (https://www.genecards.org/, accessed on 10 January 2024) [60], OMIM (https://omim.org/, accessed on 11 January 2024) [61], and PharmGKB (https://www.pharmgkb.org/, accessed on 12 January 2024) [62]. GeneCards was utilized for its comprehensive genomic and functional annotations, while OMIM provided detailed information on genetic phenotypes and Mendelian disorders. Additionally, PharmGKB was consulted to retrieve pharmacogenomic data specifically related to TF. All identified targets were subsequently merged and deduplicated to establish a consolidated set of TF-related genes for further analysis.

### 4.3. PPI and Modular Identification

PPI networks were constructed using the STRING database (https://string-db.org, accessed on 18 February 2024) [63]. The STRING platform was used to examine the intersection between targets related to BLW and TF for assessing their potential as therapeutic candidates. By processing relevant gene sets, interaction data were extracted to establish a PPI network. This network was specifically curated for Homo sapiens and utilized a medium confidence threshold of 0.4 to maintain the validity of the interactions. To evaluate the network’s architecture, Cytoscape (v3.10.2) was used to compute key topological parameters, including the degree value, closeness centrality, and betweenness centrality. Furthermore, the MCODE plugin was applied to identify highly connected clusters or central nodes within the network, using a configured degree cut-off of 2, a k-core of 2, a node score cut-off of 1.0, and a maximum depth of 100.

### 4.4. Functional and Pathway Enrichment Analysis

Functional annotation of the identified therapeutic targets was performed using DAVID Bioinformatics Resources 6.8 (https://davidbioinformatics.nih.gov/, accessed on 15 March 2024) [64]. This analysis utilized GO and KEGG pathway enrichment to characterize the biological significance of the targets. GO terms were systematically classified into BP, MF, and CC, while KEGG analysis identified the associated signaling pathways. Only pathways and GO terms exhibiting a *p*-value of less than 0.05 were considered statistically significant and selected for subsequent evaluation. To represent the findings of the GO and KEGG enrichment analyses, bubble plots illustrating signaling pathways and GO classifications were generated via the Bioinformatics online platform (http://www.bioinformatics.com.cn/, accessed on 21 April 2023). Furthermore, the complex interactions between bioactive substances, candidate targets, and associated signaling pathways were mapped and visualized using Cytoscape (v3.10.2).

### 4.5. Molecular Docking Studies

#### 4.5.1. Preparing Protein and Ligand Models

Three-dimensional (3D) crystal structures for four key human therapeutic targets, including TNF (PDB ID: 2AZ5), PTGS2 (PDB ID: 3LN1), STAT3 (PDB ID: 6NJS), and NFKB1 (PDB ID: 1NFK), were retrieved from the RCSB Protein Data Bank (http://www.rcsb.org/, accessed on 19 May 2024). These structures served as the molecular templates for subsequent docking simulations. All selected protein models possessed a resolution of 2.5 Å. During the acquisition process, the complete structural data of the proteins were obtained, along with information regarding their respective small-molecule ligands. For molecular docking, TNF utilized chain protein D [65], while PTGS2 used chain protein A [66]. The STAT3 unit contains two domains: a DNA-binding domain and a SH2 domain [67]. NFKB1 was removed from the structure p50 dimer and p50 monomers (chains A and B) [68]. The three-dimensional coordinates of the 15 bioactive compounds were retrieved from the PubChem database (https://pubchem.ncbi.nlm.nih.gov/, accessed on 19 May 2024). In the preparation phase, Discovery Studio 2021 was utilized to remove water molecules and pre-existing ligands from the protein structures, which were then exported as PDB files. Subsequently, AutoDockTools (v1.5.6) was used to facilitate the addition of hydrogen atoms, charge calculation, and the merging of nonpolar hydrogens. To finalize the docking inputs, both the target receptors and small-molecule ligands were converted into PDBQT formats. Within these files, all active bonds in the ligands were configured as rotatable to account for molecular flexibility during the simulation.

#### 4.5.2. Protein-Ligand Binding Interactions

Molecular docking simulations were conducted using AutoDock Vina 4 [69]. To validate the docking protocol, the spatial conformations of the docked ligands were compared against their original crystal-bound positions. The molecular docking simulations were performed using a setting of 100 binding modes (num_modes). Furthermore, the dimensions of the grid box and its coordinates were established based on parameters identified from prior literature reviews to ensure optimal binding site coverage. Specific grid box parameters were established to define the docking search space for each protein target. For TNF, a 40 × 40 × 40 Å cube with 1.0 Å spacing was centered at coordinates (x = −11.9784, y = 70.2727, z = 14.7429) [65]. The PTGS2 docking site utilized a 30 × 30 × 30 Å grid with 1.0 Å spacing, centered at (x = 31.724, y = −22.006, z = −17.132) [66]. For STAT3, the dimensions were set to 72 × 74 × 48 Å with a spacing of 2.7 Å, centered at (x = 7.202330, y = 31.187204, z = 20.437019) [67]. Finally, the NFKB1 docking site was defined by a 30 × 30 × 30 Å cube with a fine grid spacing of 0.3 Å, centered at (x = −1.1958, y = 9.0149, z = 19.7598) [68].

#### 4.5.3. Docking Validation

Molecular docking simulations were performed to assess the binding affinities between the identified bioactive constituents and their respective protein targets. These targets were selected based on the overlap between the four primary hub proteins and those with the most extensive interaction profiles. In terms of thermodynamic evaluation, a binding energy of less than 0 kJ/mol corresponds to spontaneous ligand-receptor association, whereas a value lower than −4.0 kJ/mol reflects a robust binding affinity. To ensure the reliability of the docking protocol, validation was performed using established inhibitors for each therapeutic target. Three-dimensional structures for thalidomide (CID: 5426) [70], celecoxib (CID: 2662) [71], ochromycinone (CID: 11808929) [72], and selinexor (CID: 71481097) [73], which inhibit TNF, PTGS2, STAT3, and NFKB1, respectively, were retrieved from PubChem and existing literature. This verification process was performed using AutoDock Vina 4, with experimental parameters identical to those used for the bioactive compounds.

### 4.6. Preparation of BLW Extract

Roots from five medicinal plants, specifically *Capparis micracantha* DC. (MSU.PH-CAP-C1), *Clerodendrum indicum* (L.) Kuntze (MSU.PH-LAM-C1), *Ficus racemosa* L. (MSU.PH-MOR-F1), *Harrisonia perforata* (Blanco) Merr. (MSU.PH-RUT-H1), and *Tiliacora triandra* Diels (MSU.PH-MEN-T1), were collected from Roi Et Province, Thailand, in October 2022. Assoc. Prof. Dr. Somsak Nualkaew conducted taxonomical identification. Voucher specimens for each species were subsequently deposited at the Pharmaceutical Chemistry and Natural Products Research Unit, Faculty of Pharmacy, Mahasarakham University, Thailand. Regarding the extraction process, the fresh roots were thoroughly washed, sliced into small pieces, and dehydrated in a hot-air oven at 45 °C for 72 h. The dried plant materials were subsequently reduced to coarse powders. To prepare the BLW formulation, equal proportions of the powdered plant materials were thoroughly blended and macerated in 70% ethanol at a 1:5 (*w*/*v*) ratio. This extraction process was maintained for three days with periodic agitation to ensure optimal solvent penetration. The resulting mixture was filtered through Whatman No. 1 paper, and the solid residue was subsequently macerated under identical conditions to maximize the recovery of bioactive constituents. The resulting filtrates were pooled and concentrated using a rotary evaporator at temperatures between 55 and 60 °C under a vacuum of 180–200 mbar. The crude extracts were combined and freeze-dried (38.3 g, 7.19% yield).

### 4.7. Chemicals and Reagents

Bergenin and obacunone were procured from Chengdu Alfa Biotechnology (Chengdu, China), and pectolinarigenin was procured from Wuhan ChemNorm Biotech (Wuhan, China), respectively. Specific compounds, including perforatic acid (Supplementary Figure S2), *O*-methyllaloptaeroxyrin (Supplementary Figure S3), and peucenin-7-methyl ether (Supplementary Figure S4), were isolated from the roots of *H. perforata*. Laboratory-grade solvents, specifically 95% commercial ethanol, were sourced from ITALMAR (Bangkok, Thailand). Analytical-grade reagents, including HPLC-grade acetonitrile and AR-grade DMSO, were supplied by RCI-Labscan (Bangkok, Thailand), while trifluoroacetic acid (99.9%) was obtained from Acros Organics (Antwerp, Belgium). Biological assay components, such as indomethacin, lipopolysaccharide (LPS) from *E. coli* 055:B5, and 3-(4,5-dimethylthiazol-2-yl)-2,5-diphenyltetrazolium bromide (MTT), were purchased from Sigma-Aldrich (St. Louis, MO, USA). Additionally, cell culture essentials, including fetal bovine serum (FBS), Penicillin-Streptomycin (P/S), Dulbecco’s Modified Eagle’s Medium (DMEM), 0.4% Trypan blue, and Trypsin-EDTA, were provided by Gibco (Waltham, MA, USA).

### 4.8. High-Performance Liquid Chromatography (HPLC) Analysis

The HPLC analysis was conducted using an Agilent 1260 Infinity II Prime HPLC system (Agilent Technologies, CA, USA). Chromatographic separation was achieved on a Luna C18(2) column (5 µm, 100 Å, 250 × 4.6 mm, Phenomenex^®^ (Phenomenex Inc. VA9525100, Torrance, CA, USA) with a constant flow rate of 0.8 mL/min. The mobile phase consisted of 0.1% (*v*/*v*) trifluoroacetic acid in water (A) and acetonitrile (B). The detection wavelength was set at 254 nm, with an injection volume of 20 µL and a total run time of 160 min. The gradient elution profile was programmed as follows: 2–10% B (0–30 min), 10–20% B (30–60 min), 20–30% B (60–85 min), 30–60% B (85–120 min), and 60–100% B (120–155 min), followed by a 5-min hold. The chromatographic peaks within the BLW extract were identified through a comparative analysis of retention times and UV spectra against established reference standards. These standards included bergenin, obacunone, perforatic acid, *O*-methylalloptaeroxylin, pectolinarigenin, and peucenin-7-methyl ether. The quantity of these marker compounds within the BLW extract was subsequently determined using their corresponding calibration curves.

### 4.9. Assessment of NO Inhibition and Cell Viability Effects of the BLW Extract and Biomarkers

The RAW 264.7 murine macrophage cell line, sourced from the American Type Culture Collection (ATCC TIB-71), was maintained in Dulbecco’s Modified Eagle Medium (DMEM), which was supplemented with 1% penicillin-streptomycin and 10% heat-inactivated fetal bovine serum (FBS). The cultures were incubated at 37 °C in a humidified atmosphere containing 5% CO_2_.

The inhibitory effect of the BLW extract and its biomarker compounds on NO production was evaluated following an established protocol [51]. First, RAW 264.7 cells were seeded into 96-well plates at a density of 1 × 10^5^ cells per well and allowed to adhere for 24 h. Subsequently, the cells were treated simultaneously with various concentrations of the samples (1–100 μg/mL) and 1 μg/mL of LPS for a further 24-h incubation period. To quantify NO levels, 100 μL of the culture supernatant was allowed to react with Griess reagent, and the absorbance was recorded at 520 nm. Indomethacin was used as a positive control, and all experiments were performed in triplicate. The percentage of NO inhibition was determined using the formula: % Inhibition = [(OD_control_ − OD_sample_)/OD_control_] × 100, where OD_sample_, is the optical density of the sample (treated in LPS-induced cells); OD_control_, is the optical density of the solvent (treated in LPS-induced cells). Half-maximal inhibitory concentration (IC_50_) values were obtained using GraphPad Prism software version 10.0.0 (Dotmatics, Boston, MA, USA).

Cytotoxicity of the BLW extract and its biomarker compounds against RAW 264.7 cells was assessed via the MTT colorimetric assay. After 24 h of sample treatment, 10 μL of MTT solution (5 mg/mL in PBS) was added to each well and incubated for 2 h. After removal of the supernatant, 75 μL of DMSO was added to solubilize the resulting formazan crystals. The absorbance of the formazan solution was then measured using a microplate reader at 570 nm. Samples causing cell viability below 70% were considered cytotoxic [52]. Percentage cell viability was calculated as follows: cell viability (%) = (OD_sample_)/OD_control_) × 100, where OD_sample_ represents the optical density of non-LPS-induced cells treated with the sample, and OD_control_ denotes the optical density of non-LPS-induced cells treated with the vehicle solvent alone.

### 4.10. Statistical Evaluation

Network pharmacology data were analyzed using the bioinformatics platforms previously specified, with a *p*-value threshold of less than 0.05 defining statistical significance. All laboratory experiments regarding chemical composition and biological activity were conducted in triplicate, and the resulting data were expressed as mean ± standard deviation (SD). To ensure the validity of the parametric tests, the datasets were verified for normal distribution using the Shapiro-Wilk test and for homogeneity of variance through Levene’s test. Comparative analysis between groups was performed using one-way analysis of variance (ANOVA), followed by Tukey’s post hoc test for multiple comparisons. For these analyses, a more stringent significance level was established at *p* < 0.01. All statistical analyses were carried out using SPSS software version 16.0.0 (SPSS Inc., Chicago, IL, USA).

## 5. Conclusions

Network pharmacology and molecular docking were used in this study to investigate the potential mechanisms underlying TF, focusing on BLW remedy as a therapeutic intervention. The results of this research revealed that BLW’s efficacy in the treatment of TF involves a complex interplay of multiple compounds, targets, and pathways. Specifically, BLW modulates key targets, including TNF, PTGS2, STAT3, and NFKB1, through the phenylalanine, arachidonic acid, and tyrosine metabolic pathways. These targets are central to processes such as infection, inflammation, proliferation, apoptosis, and chemotherapy resistance, all of which are implicated in TF. In addition, the BLW extract also exhibited potent NO inhibitory activity in (LPS)-induced RAW 264.7 macrophages with no cytotoxicity against RAW 264.7 cells. The antipyretic activity of BLW through various mechanisms may be associated with the phytochemicals of the five plants constituted BLW remedy, such as bergenin, perforatic acid, *O*-methylalloptaeroxylin, pectolinarigenin, and peucenin-7-methyl ether. These findings serve as a pharmacological foundation to provide scientific evidence for the clinical use of BLW in the treatment of TF by focusing on its anti-inflammatory actions and confirmation of previous experimental results of some genes in therapeutic targets.

## Figures and Tables

**Table 1:** Descriptions of the PPI network hub genes.

Target	Degree	Betweenness Centrality	Closeness Centrality	Type
TNF	47	0.669354	0.387608	Cytokines
PTGS2	35	0.588652	0.153506	Oxidoreductase
STAT3	26	0.532051	0.081417	Transcription
NFKB1	25	0.515527	0.044900	Transcription

**Table 2:** The results of MCODE enrichment analysis.

MCODE	Pathway	Description	Fold Enrichment
MCODE1	hsa00590	Arachidonic acid metabolism	22.6
MCODE1	hsa05143	African trypanosomiasis	16.0
MCODE1	hsa04913	Ovarian steroidogenesis	11.6
MCODE2	hsa00230	Purine metabolism	36.9
MCODE2	hsa05032	Morphine addiction	30.3
MCODE2	hsa01232	Nucleotide metabolism	18.5
MCODE3	hsa00360	Phenylalanine metabolism	120.3
MCODE3	hsa00350	Tyrosine metabolism	106.9
MCODE3	hsa00340	Histidine metabolism	87.5
MCODE4	hsa04020	Calcium signaling pathway	22.8
MCODE4	hsa04080	Neuroactive ligand-receptor interaction	19.7

**Table 3:** The binding energy of potentially bioactive compounds of BLW and their four target proteins.

Compounds	Binding Energy (ΔG*_bind_*_,_ kcal/mol)			
	TNF (PDB: 2AZ5)	PTGS2 (PDB: 3LN1)	STAT3 (PDB: 6NJS)	NFKB1 (PDB: 1NFK)
**Bioactive compounds**				
(+)-Vouacapenic acid	−8.9	−7.8	−7.5	−8.1
5,7-Dihydroxy-6-oxoheptade canoic acid	−5.1	−6.5	−4.5	−5.1
Bergapten	−6.5	−8.1	−6.0	−7.6
Bergenin	−7.3	−7.2	−7.1	−8.6
Harperamone	−6.6	−8.0	−5.9	−8.0
Hispidulin	−7.4	−9.4	−7.7	−9.3
Magnoflorine	−7.9	−8.8	−7.2	−9.5
Obacunone	−9.1	−8.8	−9.3	−11.1
*O*-Methylalloptaeroxylin	−7.4	−8.9	−6.9	−8.1
Pectolinarigenin	−7.5	−8.6	−7.1	−9.6
Perforatic acid	−7.5	−8.9	−7.2	−8.5
Perforatic acid methyl ester	−7.4	−9.3	−7.4	−7.9
Peucenin-7-methyl ether	−6.5	−8.3	−6.3	−7.9
Stigmasterol glucoside	−8.9	−8.5	−7.7	−8.5
**Standard drug**				
Thalidomide	−7.4	-	-	-
Celecoxib	-	−12.1	-	-
Ochromycinone	-	-	−8.3	-
Selinexor	-	-	-	−10.2

**Table 4:** The content of biomarkers in BLW and individual ingredient plant extracts.

Marker Compounds	Content of Biomarkers (mg/g Extract)					
	BLW	CM	CI	FR	HP	TT
Bergenin (**1**)	0.22 ± 0.00 ^a,^*	ND	ND	5.45 ± 0.01 **	ND	ND
Perforatic acid (**2**)	80.89 ± 0.00 ^b,^*	ND	ND	ND	288.39 ± 0.00 ^a,^**	ND
*O*-Methylalloptaeroxylin (**3**)	53.29 ± 0.02 ^c,^*	ND	ND	ND	216.46 ± 0.03 ^b,^**	ND
Pectolinarigenin (**4**)	2.50 ± 0.00 ^d,^*	ND	3.76 ± 0.00 *	ND	ND	ND
Peucenin-7-methyl ether (**5**)	35.03 ± 0.00 ^e^	ND	ND	ND	73.51 ± 0.00 ^c,^**	ND

Values are expressed as mean ± SD of triplicate measurements (n = 3). Different superscript letters (^a, b, c, d, e^) in the same column and different symbols (*, **) in the same row indicate significant differences between groups (*p* < 0.01). Benjalokawichian (BLW); *Capparis micracantha* (CM); *Clerodendrum indicum* (CI); *Ficus racemosa* (FR); *Harrisonia perforata* (HP); *Tiliacora triandra* (TT); not detected (ND).

## Data Availability

The original contributions presented in this study are included in the article/Supplementary Material. Further inquiries can be directed to the corresponding author.

## Supplementary Materials

The following supporting information can be downloaded at: https://www.mdpi.com/article/10.3390/ijms27062697/s1.

## Abbreviations

The following abbreviations are used in this manuscript:

BLW	Benjalokawichian
BP	Biological Process
CC	Cellular Component
COX-2	Cyclooxygenase-2
GI	Gastrointestinal
GO	Gene Ontology
iNOS	Inducible Nitric Oxide Synthase
IL1B	Interleukin-1β
IL6	Interleukin-6
KEGG	Kyoto Encyclopedia of Genes and Genomes
MCODE	Molecular Complex Detection
MF	Molecular Function
NFKB1	Nuclear Factor-kappa-B p105 Subunit
NO	Nitric Oxide
PGE_2_	Prostaglandin E2
PPI	Protein-Protein Interaction
PTGS2	Prostaglandin-Endoperoxide Synthase 2
STAT3	Signal Transducer and Activator of Transcription 3
STRING	Search Tool for the Retrieval of Interacting Genes
TF	Toxic Fever
TNF	Tumor Necrosis Factor-α
TTM	Thai Traditional Medicine
