# Supplementary material for: Integration of Network Pharmacology and Molecular Docking Together with an In Vitro Nitric Oxide Inhibition for the Insight for Antipyretic Effects of Benjalokawichian, the Thai Traditional Polyherbal Remedy
Source: Int J Mol Sci. 2026 Mar 16;27(6):2697. doi: 10.3390/ijms27062697 (PMC13026877; doi:10.3390/ijms27062697)
Supplement: Supplementary file 1 [file ijms-27-02697-s001.zip › ijms-4173270-supplementary.pdf]

Supplementary Materials

Integration of Network Pharmacology and Molecular Docking Together with an In Vitro Nitric Oxide Inhibition for the Insight for Antipyretic Effects of Benjalokawichian, the Thai Traditional Polyherbal Remedy

Chinnaphat Chaloemram [1], Ruchilak Rattarom [2], Anake Kijjoa [3] and Somsak Nualkaew [2,*]

[1] Doctor of Philosophy in Pharmacy Program, Faculty of Pharmacy, Mahasarakham University, Kantharawichai 44150, Maha Sarakham, Thailand; chinnaphat.med@gmail.com
[2] Pharmaceutical Chemistry and Natural Product Research Unit, Faculty of Pharmacy, Mahasarakham University, Kantharawichai 44150, Maha Sarakham, Thailand; rujiluk.r@msu.ac.th
[3] School of Medicine and Biomedical Sciences Abel Salazar (ICBAS) and CIIMAR, Universidade do Porto, Rua de Jorge Viterbo Ferreira 228, 4050-313 Porto, Portugal; ankijjoa@icbas.up.pt
* Correspondence: somsak.n@msu.ac.th; Tel.: +66-85-137-6763

Table S1. ADME analysis of 32 anti-inflammatory bioactive compounds in BLW.

Anti-inflammatory bioactive compounds in MHR	GI absorption	Drug likeness
(+)-Vouacapenic acid	High	Yes
5, 7-Dihydroxy-6-oxoheptadecanoic acid	High	Yes
Bergapten	High	Yes
Bergenin	High	Yes
Chlorogenic acid	Low	No
Cycloartenol	Low	No
Daucosterol	Low	No
Friedelin	Low	No
Harperamone	High	Yes
Harperfolide	Low	No
Harrisolanol A	High	Yes
Harrisonin	Low	No
Hispidulin	High	Yes
Lupeol	Low	No
Lupeol acetate	Low	No
Magnoflorine	High	Yes
Obacunone	High	Yes
O-Methylalloptaeroxylin	High	Yes
Pectolinarigenin	High	Yes
Perforatic acid	High	Yes
Perforatic acid methyl ester	High	Yes
Peucenin-7-methyl ester	High	Yes
Racemosic acid	Low	No
Rutin	Low	No

Stachydrine	Low	No
Stigmasterol	Low	No
Stigmasterol glucoside	High	Yes
Taraxerol	Low	No
α-Amyrin	Low	No
α-Amyrin acetate	Low	No
β-Amyrin	Low	No
β-Sitosterol	Low	No

Table S2. Table S2: The 495 targets associated with 15 bioactive compounds.

Anti-inflammatory compounds in MHR	Predicted targets
(+)-Vouacapenic acid	TBXAS1, POLA1, PIK3CA, PIK3R1, HSD11B1, TBXA2R, PTGES2, PTGDR2, PTGDR, PTGES, MME, MDM2, PSEN2, PSENEN, NCSTN, APH1A, PSEN1, APH1B, PTGER4, PTGER2, PPARG, PTGER3, PTGS2, ALOX5, PTGS1, F2R, SLC22A12, ACE, PTGER1, PTGIR, FDFT1
5, 7-Dihydroxy-6-oxoheptadecanoic acid	PTGER2, PTGFR, FFAR1, SLC22A6, PPARA, PPARD, CHRNA7, HMGCR, G6PD, FDFT1, FNTA, FNTB, FABP4, FABP3, FABP5, NR1H4, AKR1B10, HSD11B1, PTGER1, PTGER4, PTGIR, UGT2B7, GPBAR1, HAO1, SERPINA6, SHBG, HSD17B3, GABBR1, NPC1L1, PTGER3, PTGDR, VDR, FABP2, PDE6D, MME, CDC25A, GABRA2, GABRB2, GABRG2, TBXAS1, KDM2A, EDNRA, MAPK14, MAPK10, MMEL1, ITGB1, ITGA4, GCGR, GIPR, CYP2C9, CA2, CTSA, CASP3, CASP6, CASP7, CASP8, CASP1, PTGDR2, EDNRB, PDE5A, EGLN1, P2RY12, P2RX3, PHF8, GSK3B, GSK3A, PGR, BACE1, SLC5A2, PLA2G4B, TBXA2R, SCN9A, KDM5C, MMP1, THRA, THRB, DUSP23, DUSP15, PTPN22, PTPN13, PTPN12, PTPN11, PTPN9, GLP1R, TYMS, PDE4B, DGAT1, IDE, AGTR1, APP, CSNK2A1, ITGAV, ITGB3, EP300, SCN10A, FOLH1, SOAT1, F5, CYP19A1, TNF, SLC6A1, MMP9, MMP2, MMP8
Bergapten	CBR1, KCNA5, KCNA3, CA12, CA9, CA7, CA13, CA1, ALOX5, ACHE, CYP1A2, CA6, CA14, CA4, CA5A, XDH, SRD5A1, CA5B, AKR1C3, AKR1C1, BACE1, MAOA, ESR2, PARP1, PARP2, NFKB1, GPR35, AKR1B1, CCND1, CDK4, PDGFRB, FLT4, INSR, PTK2, PLK1, MET, PLK4, TEK, MAP3K8, BRAF, EPHB4, HSPA1A, NUAK1, SQLE, FGR, LYN
Bergenin	F10, PNP, ADA, GSK3B, ADORA2A, ADORA3, ADK, CA14, TDP1, TYMP, GAPDH, MMP3, MMP9, MMP1, ADAM17
Harperamone	BCL2, RET, MAPK1, EPHB4, MCL1, HSP90AA1, ABCC9, MMP3, MMP13, MMP1, ADAM17, CA5A, PSEN2, PSENEN, NCSTN, APH1A, PSEN1, APH1B, PRKCB, GABRB3, GABRA3, GABRG2, GABRB3, GABRG2, GABRA1, GABRB3, GABRG2, GABRA5, GABRA2, GABRB3, GABRG2, GABRG2, GABRB3, GABRA6, MAP2K1, MPO, PDE4B, TTK, MAPK14, JAK1, CHEK1, TKT, PDE2A, PRKDC, PDE10A, BRD4, BRD2, BRD3, MME, MMP14, EIF2AK3, ABCB1, COMT, MTOR, PIK3CD, PIK3CB, MMP8, TNK2, PIK3C2B, NTRK1, KIF11, PDE3A, NQO2, MMP7, ERN1, CREBBP, PDE5A, ADA, NR3C1, CXCR2, PTAFR, CXCR1, HSD11B1, PDE7A, CYP17A1, TLR8, TLR7, ALOX5, OPRK1, HMGCR, EDNRA, CACNA1C, SRC, GRM5, HCK, SCN9A, PAK1, CASP8, CASP1, CYP2D6, PRKCG,

	CX3CR1, CDC7, KDM1A, ASAH1, IMPDH2, EWS-Fli1, CCR5, KCNH2, PI4KB, MDM2, MAPKAPK2, TYMP, BAZ2B, FCER2, BAZ2A, CYSLTR1, ELAVL1, EDNRB, ADRA2A, GYS1, F2, PLEC, MAP3K12, PSEN1
Harrisolanol A	-
Hispidulin	PIM1, ADORA1, ADORA2A, AKR1B1, PTPRS, KIT, OPRD1, FLT3, ESR2, AMY1A, GRK6, NOS2, NOX4, ESR1, HSD17B1, EGFR, CYP1B1, CDK5R1, CDK5, APP, TERT, ABCC1, CBR1, AR, XDH, PFKFB3, ALOX5, KDM4E, ALOX15, CDK1, ALOX12, ABCG2, HSD17B2, ADORA3, ABCB1, TNKS2, TNKS, MAOA, CYP19A1, CCNB3, CDK1, CCNB1, CCNB2, ACHE, PTGS2, CDK6, SYK, GSK3B, TTR, CSNK2A1, CFTR, AKR1B10, CA2, CA7, CA12, CA4, PLG, PLA2G2A, SLC22A12, CA1, CA9, F2, NAE1, ODC1, MCL1, GLO1, PARP1, MMP9, MMP2, MMP12, CD38, TOP1, ARG1, BACE1, TYR, AHR, ESRRA, CALM1, MAOB, IGF1R, ST6GAL1, OPRM1, LCK, SIGMAR1, DRD2, CYP1A1, CYP1A2, PTPN1, GPR35, DAPK1, MPG, PIK3CG, AURKB, SRC, PTK2, KDR, PLK1, PKN1, MET, NEK2, ALK, AKT1, NEK6, AXL
Magnoflorine	DRD2, CHRNA4, CHRNB2, DRD3, DRD1, HTR1A, HTR7, HTR6, HTR2B, PTPRCAP, HTR2A, ADRA1D, TH, DRD4, HTR5A, ADRA1A, HTR1D, ADRB1, SLC6A3, DRD5, HTR2C, OPRM1, KCNH2, ADRA1B, TSPO, SLC6A4, HRH2, ADRA2A, ADRA2C, ADRA2B, HTR1B, PTGES, CDK5R1, CDK5, DYRK1A, MAPK14, RBP4, TRPC6, TRPC3, ALOX15, CYP19A1, IRAK4, ADRB2, JAK3, ERN1, STAT3, KDR, PIM1, MMP9, MMP2, MAPKAPK2, CDK1, PPARG, CCKBR, PPP5C, CCNB3, CDK1, CCNB1, CCNB2, GSK3B, NR3C2, EGFR, MGLL, ALOX12, AKR1B1, CASP3, MMP1, AURKA, SIGMAR1, SLC9A1, PREP, HSD17B3, RPS6KB1, FAP, RORC, CDK2, CCNA1, CCNA2, MAP2K1, AR, GABRA1, EPHX2, ADORA2A, GABRA5, MIF, HPGD, FLT3, MAPK3, MAPK1, CSF1R, PRKCG, LIPG, CCNE1, CDK2, HSD17B2, CCNE1, CDK3, PGR, DBF4, CDC7, ADAMTS5, PDGFRA, PDGFRB, SRC, MMP3, CYP17A1, FGFR1, EIF2AK2, F3, RPS6KA3, PDPK1
Obacunone	OPRK1, OPRM1, OPRD1, MIF, P2RX3, AOC3, BACE2, BACE1, CTSK, CTSS, CTSV, MAPK1, P2RX7, PSEN2, PSENEN, NCSTN, APH1A, PSEN1, APH1B, PDE7A, DRD2, HTR2A, PLA2G2A, PIM1, FBP1, PIM2, HMGCR, CDK5R1, CDK5, DYRK1A, HTR7, EPHX2, CTSL, PFKFB3, CCR1, CYP19A1, AVPR1A, C5AR1, ITK, CDK9, CDK2, CCNA1, CCNA2, PARP1, MDM2, SCN9A, EPHX1, OXTR, CFD, CNR1, SERPINA6, CNR2, MAPK14, HDAC1, SHBG, HDAC4, BRS3, LRRK2, MAOB, CCNE1, CDK2, SCN5A, NR1I2, CLK4, CLK1, CLK2, HSD11B1, AKR1C3, DYRK1B, PSMB8, SMYD2, PDE9A, F2R, CHRM2, CHRM1, CHRM3, SIGMAR1, MAP2K1, LIPE, NAMPT, ADRA2B, DRD3, ADORA3, KDR, CDK1, MET, FASN, TAAR1
O-Methylalloptaeroxylin	PDE4D, CYP1A1, CYP1A2, CYP1B1, CA7, CA1, CA12, CA9, ACHE, JAK2, MTNR1B, AR, TNFRSF1A, TRPA1, FLT3, PRKDC, ABCG2, RARA, IDO1, CA13, ADAMTS5, NOTUM, MAPK10, GABRB3, GABRG2, GABRA5, KCNA5, CCR8, MAPK14, TGFBR1, ACVR1B, CYP19A1, PARP1, NQO2, HSD11B1, PDE10A, HCRTR2, HCRTR1, CTSK, CTSS, CTSL, CYP11B1, CYP11B2, CYP17A1, P2RX7, SCN10A, CNR1, CNR2, DHODH, GAPDH, CCNC, CDK8, CCNE2, CDK2, CCNE1, GRM5, GSK3B, DYRK1B, BCHE, PDE3A, PDE7A, BDKRB2, PREP, HDAC3, HDAC6, JAK3, JAK1, HDAC1, TYK2, PARP2, MAPK8, PIM2, FAP, PDE2A, GUSB, ICAM1, NOS1, SELE,

	NOS2, HSP90AA1, GRM4, IKBKB, CCND3, CCND1, CDK4, CCND2, CDK9, CCNT1, CDK2, VCAM1, PIK3CA, HTR1A, DRD2, PDE4A, SRC, PDE4B, CHUK, PTGER2, TGM2, MAP2K1, BAZ2B, SYK, BAZ2A, MET, GABRA1, GABRB2, GABRG2, ADORA2B, CDK7, HTR7, HTR6
Pectolinarigenin	KIT, OPRD1, AKR1B1, PIM1, ADORA1, ADORA2A, ADORA3, FLT3, CYP1B1, NOS2, NOX4, HSD17B1, ESR1, ESR2, ABCB1, ALOX15, ALOX12, ABCC1, APP, PTGS2, CFTR, GRK6, PTPRS, XDH, GSK3B, PLA2G2A, CA2, CA4, CDK1, CA9, HSD17B2, CYP19A1, MMP9, MMP2, OPRM1, CA7, CA12, CA1, CDK5R1 CDK5, MAOA, NAE1, AMY1A, ODC1, CCNB3 CDK1 CCNB1 CCNB2, CDK6, LCK, PIK3CG, ABCG2, ALOX5, KDM4E, CBR1, SIRT1, BACE1, TNKS2, TNKS, NTRK2, BCHE, CSNK2A1, ACHE, MCL1, TERT, EGFR, PLG, CALM1, MAOB, SYK, TTR, AKR1B10, ST6GAL1, CA6, AKT1, ALK, SLC22A12, SIGMAR1, AR, CYP1A1, CYP1A2, GLO1, ARG1, PFKFB3, IKBKB, MET, PARP1, MMP12, CD38, TOP1, HSP90B1, F2, AVPR2, CXCR1, GPR35, ALDH2, IGF1R, PPARG, PTPN1, TYR, AHR, ESRRA, HSP90AB1, AURKB
Perforatic acid	CYP1A1, CYP1A2, CYP1B1, PTGDR2, KDM5B, NR4A1, AKR1C3, PPARA, PPARD, PDE4D, SLC22A12, MKNK2, MKNK1, KDM4E, KDM3A, KDM2A, KDM5C, CNR2, METAP2, KDM4C, KDM2B, AKR1C2, CCKAR, SLC16A3, MAPK8, MAP3K8, PLEC, ITGB1 ITGA5, ITGB1 ITGA2, ITGB1 ITGA1, AKR1A1, CA1, ICAM1, SELE, SORT1, PTGER4, GRIK1, KMO, PLA2G2A, KDM4D, FABP4, ERCC5, FEN1, NR1H4, KDM6B, PRKDC, GYS1, CASP1, PIM1, ITGAV, DYRK2, PIM2, CAMKK1, CAMKK2, PIM3, TBXAS1, CDC25B, ECE1, P4HTM, TTR, MAPK1, CTSA, SCN10A, ACE, ITGAV ITGB3, MME, MMP2, PIK3CA, AURKA, PTGS1, ACE2, FFAR1, ADAMTS5, RHOA, HAO1, HSPA1A, CREBBP, CDC25A, MCL1, ALOX5AP, CDK2 CCNA1 CCNA2, CTSS, PLK1, ABL1, TDO2, IDO1, CPA1, CSNK2A1, ABAT, ITGB1 ITGA4, PFKFB3, PAM, PFKFB4, PFKFB4 PFKFB3, ERBB2, THRA, THRB, ITGAL ICAM1 ITGB2, PDE10A, BCL2
Perforatic acid methyl ester	MAPK8, KDM4E, KDM5C, KDM4A, KDM4D, KDM4C, CDK2 CCNA1 CCNA2, CDK2, PDE4D, PIM1, PIM2, MTNR1A, MTNR1B, NQO2, P2RX7, TAAR1, FLT3, CDK9 CCNT1, PIK3CA, ADAMTS5, CDK4, HSD11B1, FAP, CXCR2, ADORA2B, SYK, MAPK14, CSNK1D, GRM5, ADORA2A, PREP, PLCG2, JAK2, PDE10A, SRC, IKBKE, TBK1, HTR2C, LRRK2, KDM5B, CCNE2 CDK2 CCNE1, PDE4B, MET, IKBKB, ELANE, CA6, CA14, CA4, CTSS, SCN10A, TERT, CA7, CA12, CA9, CDC7, PIK3CD, PRKDC, PIK3CB, HCK, PIK3CG, PI4KB, EPHB4, FLT4, RET, RPS6KA1, PDE2A, PDE11A, KDR, MAP2K2, PDE7A, MAP2K1, FGR, RPS6KB1, TLR4, ABL1, CA2, AURKB, MTOR, MAPK9, JUN, CDK5R1 CDK5, HTR2A, GSK3B, GSK3A, IRAK4, PRMT3, RARA, CYP1A1, NOX4, TBXAS1, GRM4, KDM4B, KAT2B, PARP1, BCAT2, PARP2, CA1, PDGFRA PDGFRB, JAK3, MAPK10
Peucenin-7-methyl ester	NFKB1, PPARG, PDE4D, ALOX15, FASN, PTGS1, PTGS2, ALOX12, ABCB1, ABCG2, IDH1, MAOA, EGLN1, RELA, CNOT7, GRM5
Stigmasterol glucoside	IL2, STAT3, BCL2L1, PSEN2, PSENEN, NCSTN, APH1A, PSEN1, APH1B, PTAFR, PTPN1, PFKFB3, PTPN2, CDC25B, ACP1, F2, PPM1B, PPP1CC, PPP2CA, PPP2R5A, MET, HSD11B2, HSD11B1, S1PR3, S1PR1, DRD4, PPARA, GPR119

Table S3. The targets associated with TF.

PharmKrb-PF (97)	Genecards-PF (8,031)	OMIM-PF (11)
AHR, AKR1C1, ALOX15, ALOX5, AR, BMP1, BMP4, CTSG, CTSK, CTSL, CTSS, CYP27B1, EPHX1, F5, FGFR1, FPR1, GSTM1, HCAR2, HDAC1, HDAC10, HDAC2, HDAC3, HDAC4, HDAC6, HDAC9, HPGDS, IL1B, IL2, IL6, LTA4H, MAP2K4, MAP3K14, MAP3K8, MB, MIF, MPO, NFKB1, NOS2, NOX4, NR1D1, NR4A1, OXTR, P2RY12, PGR, PKN1, PLA2G2A, PLEC, PLG, PTAFR, PTGES, PTGES2, PTGS1, PTGS2, RARA, SELE, STAT3, STAT5A, TERT, THRA, TNF, TSPO, TTR, VEGFC, ADA, ADK, ADORA1, ADORA2A, ADORA2B, ADORA3, AKR1B10, CFD, CNR1, CNR2, DHFR2, MGLL, NAMPT, PDE10A, PDE11A, PDE1B, PDE2A, PDE3B, PDE4A, PDE4B, PDE4D, PDE5A, PDE7A, PDE9A, PNP, SLC22A12, TPMT, TYMP, XDH, INSR, PRKCA, PRKCB,	ACCS, CNP, OAS1, OASL, HMGCL, HMGCR, HMGCS2, BDH2, HIBCH, KDSR, OXCT1, LOC107303338, LOC107303340, PAPSS2, PDPK1, ABAT, HOGA1, HPD, XRN1, ATIC, ALAS2, AZI2, HTR1A, HTR1D, HTR2A, HTR2C, HTR3A, HTR3B, HTR3C, HTR4, MTRR, NT5DC2, NT5E, NT5C1A, NT5C1B, NT5C3A, OPLAH, PFKFB1, PTS, OGG1, A2ML1-AS1, AHI1, ABHD16A, ABHD5, LOC107980440, ABI1, ABL1, LOC112679198, ABO, AIM2, LOC117134593, ACHE, ACAT1, ACAA2, ASMT, ASCL1, ACP1, ACP5, ASIC1, ANP32A, ACO2, ACOD1, ACR, ACRV1, ACTA2-AS1, ACTA1, ACTA2, ACTC1, ACTB, ABRA, ACTL9, ACTR3, ARPC1B, ARPC2, ACTN4, ATF6, ATF6B, AICDA, AHSA1, ACVRL1, ACVR1, ARC, APEH, ACBD3, ACAD8, ACADL, ACADM, ACADS, ACADSB, ACADVL, ACSF3, ACSL4, ACSM6, ACSS1, ACOT11, ACOT12, ACOT13, ACOT8, AGK, AOAH, ADAMDEC1, ADAM15, ADAM17, ADAM22, ADAM23, ADAM28, ADAM33, ADAM7, ADAMTS13, ADAMTS4, ADAM7-AS2, ADAM7-AS1, ADAMTSL1, AP3B1, AP1S3, AP5M1, AP3D1, AP4E1, ADD3, APRT, ADORA1, ADORA2A, ADORA2B, ADORA3, ADA2, LOC107303343, ADAR, ADARB1, ADARB2, ADAT3, ADK, AMPD1, AHCY, AMD1, ADCY9, ADCY10, ADCYAP1, AK2, ADSL, ADSS2, ADGRA2, ADGRE2, ADGRE5, ADGRL2, ADGRV1, APMAP, ADIPOR1, ADIPOQ, ARF1, ARFGEF1, ARL6, ARL11, ARL2BP, ARL5B, ARFRP1, ADPRH, ADRA1A, ADRA1D, ADRA2A, ADRA2B, ADRA2C, ADRB2, ADM, AGER, AVIL, AFDN, AFM, AFAP1-AS1, AFG3L2, AGMX2, AGBL3, ARMS2, ACAN, AGRN, AHNAK, AKAP3, AKAP14, AKAP17A, AKIP1, AKIRIN2, AKT1, AGXT, ANPEP, AARS1, AARS2, ALB, ADH1A, ADH1B, ADH1C, ADH4, ADH5, ADH7, ADHFE1, ALDH4A1, ALDH16A1, ALDH18A1, ALDH2, AOX1, AKR1A1, AKR1B1, AKR1B10, AKR1C1, ALDOA, AFF2, ALG1, ALG11, ALG12, ALG13, ALG14, ALG2, ALG3, ALG5, ALG6, ALG8, ALK, ACER3, ALPL, ALPG, ALPP, ALKBH1, ALKBH3, ALKBH5, AGMO, ALRH, AIF1, AIF1L, ALMS1, A4GALT, AHSG, AFP, GAA, AHSP, ALPK1, MGAT5, AMBP, A2M, A2ML1, FUCA1, IDUA, AMACR, NAGA, ALX3, ALYREF, AMBN, AMELX, AOC1, AOC4P, AIMP1, ACY1, AASS, ALAD, AMT, AMPH, AREG, AGL, APP, APBB1IP, APCS, ANAPC13, AR, ANG, AMOT, ANGPT2, ANGPTL6, ACE, AGTR1, AGT, ANLN, ANKH, ANK1, ANKFY1, ANKK1, ANKLE2, ANKMY1, ASB2, ANKS1A, ANKRD12, ANKRD17, ANKRD24, ANKRD27, ANKRD42, ANKRD46, ANKRD49, ANKRD55, ANKRD60, ANKRD61, ANKRD11, ANKRD26, ASZ1, ANXA1, ANXA10, ANXA11, ANXA2, ANXA4, ANXA5, ANXA6, ANXA8, ANO1, ANO10, ANOS1, AMN1, AMH, ATOX1, AIRN, AZIN1, ANTXR2, AAK1, AP4B1-AS1, APCDD1, AMER1, APC, APOC4-APOC2, APOA1, APOA4, APOE, APOBEC1, APOBEC3G, APOBEC3H, APOC1, APOC2, APOC3, APOL1, APOL2, APOL3, APOL4, AATF, AATK, AIFM1, API5, APAF1, APTX, APLF, APEX1, AQP5-AS1, AQP1, AQP10, AQP11, AQP12A, AQP2, AQP3, AQR, ALOX12B, ALOX12, ALOX15, ALOX5, ALOX5AP, BRAF, ARCN1, ACAP3, AGFG1, ARG1, RSRC1, AVP, AVPR1A, AVPR1B, AVPR2, RERE, ASL, ASS1, RNPEP, ATE1, RARS1, RARS2, AGO2, AGO1, ARHGEF26-AS1, ARX, ARMC12, ARMC5, ARRB2, ARV1, AHR, AIP, AIPL1, AHRR, AADACL3, ARSD, ARSJ, ASGR1, ASPG, ASNS, NARS1, NARS2, ASPH, ASPDH, DRICH1, ASPRV1, ASPA, AGA, DARS2, ASPSCR1, ASRT3, ASTN2, ASXL1, LOC130058658, LOC130058885, LOC130058886, LOC130059053,	IL1B, IL2, IL6, NFKB1, NOS2, NOX4, PLA2G2A, PTGES, PTGES2, STAT3, TNF

PRKCD, PRKCE, PRKCG, PTK2B, TRPA1, TRPM8, TRPV3, TYR, CPT2, FADS1, FASN, TKT	LOC130059054, LOC129929080, LOC130059663, LOC130059892, LOC130059980, LOC130060043, LOC129929085, LOC130060959, LOC129929097, LOC129930930, LOC129929101, LOC129933155, LOC129934769, LOC129935044, LOC129935045, LOC129935046, LOC129935047, LOC129935405, LOC129936244, LOC129996027, LOC129929542, LOC130068093, LOC130068308, LOC130003807, LOC130006234, LOC130007232, LOC130007233, LOC130007242, LOC129930068, LOC129930245, LOC130063979, LOC130064021, LOC130064417, LOC129934333, LOC129935043, LOC129931648, LOC129936806, LOC129938041, LOC129992118, LOC129992304, LOC129997612, LOC129999940, LOC130001862, LOC130002910, LOC129929052, LOC130003418, LOC129929076, LOC129929077, LOC129929078, LOC130004025, LOC130004293, LOC129929079, LOC129929081, LOC130004578, LOC129929082, LOC129929083, LOC129929084, LOC129929086, LOC129929087, LOC129929088, LOC129929089, LOC129929090, LOC129929091, LOC129929092, LOC130006561, LOC129929093, LOC130006765, LOC129929094, LOC129929095, LOC129929096, LOC129929098, LOC129929099, LOC129929100, LOC129929102, LOC130008987, LOC129929103, LOC129929104, LOC129929105, LOC129929106, LOC130009810, LOC129929107, LOC129929108, LOC129929109, LOC129929110, LOC130055692, LOC129929111, LOC130055850, LOC130056217, LOC130058158, LOC130058479, LOC130058887, LOC130059760, LOC130059979, LOC130059981, LOC130060040, LOC130060041, LOC129930561, ATXN1, ATXN2L, ATXN10, ATCAY, ATL3, ATM, ATOH7, ABCB1, ABCA12, ABCC11, ABCB6, ABCC1, ABCC4, ABCG2, ATP5F1B, ATP5MC3, ATP5MK, ATPAF1, ATP2B1-AS1, ATP6V0E2-AS1, ATP13A1, ATP13A2, ATP7A, ATP7B, ATAD1, ATAD3C, ATP6AP1, ATP6V0A2, ATP6V0A4, ATP6V1A, ATP6V1B1, ATP6V1B2, ATP6V1E1, ATP6V1G2, ATP12A, ATP4A, ATP1A2, ATP1B1, ATP8B1, ATP2B4, ATP2A1, ATP2C2, ATRIP, ATR, ARID1A, ARID1B, ARID2, ARID3B, ARID4A, ARID4B, ARID5B, ATRIP-TREX1, ATN1, ATRX, ATXN8OS, ACKR1, ACKR2, AUH, AUP1, AURKA, AMFR, AIRE, ATG12, ATG16L1, ATG2A, ATG2B, ATG5, ATG9A, AXIN1, AXL, AXDND1, LOC106128902, LOC108004538, AZF1, AZU1, BTLA, BLNK, BCAP31, BANK1, B3GAT1-DT, BAALC, BABAM2-AS1, BPI, BIRC3, BCL7A, BCL7B, BCL7C, BANF1, BAG3, BAIAP2-DT, BAIAP2, BBS2, BSND, BPY2, BPY2B, BPY2C, BMAL1, BHLHE22, BZW1, BLZF1, BSG, BBX, BCAS2P2, BCL10, BCL11A, BAK1, BCL2, BAD, BAX, BBC3, BOK, BNIP3, BCL2L1, BCL2L10, BCL2L11, BCL2L12, BCL2A1, BCL3, BCOR, BCORL1, BCL6, BCL6B, BCL9, BCR, LOC107963955, LOC107963951, BCS1L, BDNF-AS, BFSP2, BECN1, BEST1, BGLT3, B4GALT1, B3GAT1, MGAT3, B2M, BCO1, LOC110006319, HBB-LCR, BHMT, BACE1, BTRC, UPB1, BID, BICC1, BICRAL, BLVRA, BLOC1S6, BTD, BIVM-ERCC5, BLK, BLM, BLOC1S5-TXNDC5, BMI1, BAMBI, BRINP3, BMS1P20, BMX, BOLA3, BRS3, BGLAP, BST2, BMP6, BMP10, BMPR1B, BMPR2, BOLL, BPIFB1, BDKRB1, BDKRB2, BANCR, BASP1, BCYRN1, BDNF, BCAS1, BCKDHA, BCKDHB, BRAT1, BAP1, BARD1, BRCA1, BRIP1, BRCA2, BICRA, LOC126805577, LOC126805969, LOC126805994, LOC126805749, LOC126860933, LOC126860970, LOC126861339, LOC126861360, LOC126861451, LOC126861834, LOC126862860, LOC126862902,	

	LOC126862841, LOC126806396, LOC126806658, LOC126806659, LOC126859963, LOC126860368, LOC126860369, LOC126863256, BCAR4, BLTP3A, BIN1, BROX, BAHD1, BAZ1A, BRWD1, BRD2, BRDT, BTK, BACH2, BUB1B, BTNL2, BTN2A1, BTN3A2, BCHE, BYSL, C1QTNF12, C2CD6, C9orf72, CA3-AS1, CBARP, CACNA1G-AS1, CDH1, CDH11, CDH13, CDH16, CDH17, CDH18, CDH26, CELSR1, CDH23, CDHR3, CALB1, CALCRL, CALCA, CALCB, CANT1, CIB3, CALCOCO2, CABP1, CACFD1, CASR, CACNA2D1, CACNB4, CACNG1, CACNA1A, CAMK2A, CAMK2D, CAMK2G, CAMK4, CASK, CACYBP, CALD1, CALM3, CAMTA1, CALML4, CANX, CAPN5, CAST, CALR, CASQ1, CREB1, CREB3L1, CREM, CAGE1, CASC11, CASC15, CASC8, CTAG1B, CT55, CT83, CT45A1, CT47A1, CT47A10, CT47A11, CT47A12, CT47A2, CT47A3, CT47A5, CT47A6, CT47A7, CT47A8, CT47A9, CT70, CT75, CNR1, CMTR1, CIC, CAPZB, CARMIL2, CPS1, CAD, CHST4, CA2, CA10, CA12, CA13, CA14, CA5A, CA5B, CBR1, CEL, CES1, CPA1, CPA2, CPA3, CPA6, CPB1, CPB2, CPQ, CPN1, CARD11-AS1, CARD8-AS1, CARMN, CRLS1, CRMA, CTF1, CLCF1, CPT1A, CPT2, CRTAP, CILP, COMP, CSN1S1, CSN3, CSNK2A1, CSNK1D, CSNK1E, CSNK1G2, CSNK2B, CLPX, CFLAR, CASP1, CASP10, CASP14, CASP16P, CARD11, CARD14, CARD9, CASTOR3P, CASZ1, CECR7, CAT, COMT, CTNNA1, CTNNB1, CTNNBIP1, CTNND1, CAMP, CTSB, CATSPER2, CDX2, CAVIN3, CAV2, CBFA2T3, CBL, CBLL1, CITED1, CBR3-AS1, CXCL8, CCL11, CXCL13, CCL17, CCL18, CCL19, CCL20, CCL21, CCL22, CCL24, CCL25, CCL26, CCL27, CCL3L1, CCR5, CCR10, CCRL2, CEBPA, CEBPB, CEBPD, CEBPE, CEBPZ, CTCF, CTCFL, CCDC169-SOHLH2, CCDC26, CCL15-CCL14, CCNO-DT, CNOT9, CCR5AS, CCZ1, CD101, CD14, CD163, CD164, CD177, CD180, CD19, CD1A, CD1B, CD1C, CD1D, CD1E, CD2AP, CD2-LCR, CD2, CD200, CD207, CD209, LOC117307477, CD22, CD226, CD24, CD244, CD247, CD248, CD27-AS1, CD27, CD274, CD276, CD28, CD3D, CD3E, CD300LD, CD300LD-AS1, CD320, CD33, CD34, CD36, CD37, CD38, CD4, CD40LG, CD40, CD44, CD46, CD47, CD48, CD5, CD5L, CD52, CD55, CD58, CD59, CD6, CD63, CD68, CD69, CD7, CD70, CD72, CD74, CD79A, CD79B, CD8A, CD8B, CD80, CD81-AS1, CD81, CD82, CD83, CD84, CD86, CD9, CD93, CD96, CD99, CD99L2, CDIN1, CDC42EP4, ARHGEF9, CDK5RAP1, CDK6-AS1, LOC126805874, LOC126805890, LOC126861013, LOC126860971, LOC126861452, LOC126861664, LOC126862123, LOC126862264, LOC126862361, LOC126862586, LOC126862865, LOC126806147, LOC126863160, LOC126860303, LOC126863253, LOC125467768, CIZ1, CDKN2B-AS1, CDIPT, CRPPA, CDR1-AS, CEACAM5, LOC110599576, CADM1, CAPRIN1, CIDEC, CDC14A, CDC14B, CDC25A, CDC25C, CDC27, CDC37, CDC40, CDC42, CDC6, CDC73, CCAR1, CCN2, CRABP1, CEMP1, CETN2, CENPB, CENATAC, CEP104, CEP120, CEP290, CEP70, CEP85L, CERS3, CERT1, CDR1, CRBN, CP, CCDST, CCEPR, CFTR, CFTR-AS1, LOC111674475, LOC113633877, LOC113664106, LOC111674477, LOC111674463, CCT7, CLC, CHMP2B, CHMP6, CHEK2, ENSG00000235775, CBY1, CHIT1, CHI3L1, CHIA, CHKB-CPT1B, CLCA1, CLIC1, CLCN2, CLCNKA, CLCNKB, CHM, PSC, CCK, CCKAR, CH25H, CETP, CHKA, CHKB, CHAT, CHRM2, CHRNA1, CHRNB2, CHRND, CHRNE, CHRNG, CSGALNACT1, CSPG4, CNMD, CHRDL1, CGB3, CSH2, CHRFAM7A, CHAF1B, CDT1, CHTOP, CBX5, CHD2, CDY1, CDY1B, CDY2A, CHGA, C1orf141, C1orf159, C11orf65, C12orf75, C14orf132, DEL15Q11.2, DEL15Q13.3,	

	C16orf92, C17orf107, DEL17Q12, C19orf12, C19orf53, C2orf69, C2orf88, C20orf204, C21orf91, C22orf39, C6orf118, C6orf132, C6orf47, C7orf33, LOC109504728, CSE1L, CMA1, CTRC, CTRL, CELA2A, CTRB1, CTRB2, CFAP410, CFAP43, CFAP45, CFAP47, CFAP52, CFAP54, CFAP61, CFAP91, CIMIP1, CNTF, CNTFR, CROCC, CPLANE1, CILK1, CATIP, CS, CLRN2, CLSPN, CIITA, CLTC, CLDN2, CLDN10, CLDN11, CLDN14, CLDN16, CLDN18, CLDN19, LOC106501713, CPSF4, CLN3, CLN5, CLN6, CLN8, CLOCK, CLPB, CLPTM1L, CLU, CNKSR3, F2R, F2, F3, F9, F5, F7, F8, F8A1, F12, F13B, CBLIF, COCH, CDAN1, COASY, COQ2, COQ4, COQ6, COQ8A, COQ8B, COQ9, CCHCR1, CC2D2A, CCDC40, CCDC102A, CCDC115, CCDC120, CCDC122, CCDC167, CCDC177, CCDC180, CCDC22, CCDC34, CCDC6, CCDC62, CCDC68, CCDC78, CCDC83, CCDC85A, CCDC85B, CCDC85C, CHCHD10, CHCHD5, COIL, CLPS, CCBE1, COLQ, COL1A1, COL2A1, COL3A1, COL4A3, COL6A5, COL7A1, COL11A2, COL13A1, COL17A1, COL18A1, COL20A1, COL25A1, COL26A1, COL27A1, COLEC10, CLTRN, CCAT1, CSF3, CSF3R, CSF2RA, CRNDE, COMMD3-BMI1, CERNA2, CERNA3, C1QB, C1QBP, C1R, C1S, C2, C3, C3AR1, CR1, CR2, C4A, C4B, C5, C5AR1, C6, C7, C8B, C9, C4BPA, C4B_2, CFH, CFHR1, CFP, CPLX1, CHUK, COG5, CRX, CNKSR2, CNTN1, CNTNAP2, COPS3, COPS7A, COPA, COPB2, COPE, COPG1, CPNE5, COMMD1, CPOX, CBFB, CIR1, CDB2, CDSN, CNIH1, CORO1A, CORO2A, CTTN, CTTNBP2, CRH, CRHBP, CRHR1, CORT, CPS1-IT1, CRP, CKB, CKMT1A, CKMT1B, CKMT2, CKM, CREBBP, LOC117038795, LOC115801415, CRKL, CRK, CRB2, CFC1, CRY1, CRYAB, CRYGC, LOC111188156, CSMD2-AS1, CTC1, CTAGE3P, CTAGE4, CTDP1, CSK, CTNS-AS1, CTPS1, CTR9, CTXN2-AS1, CLEC11A, CLEC16A, CLEC17A, CLEC5A, CLEC6A, CLEC7A, CLEC4M, CLEC12A, CLEC18A, CSMD1, CDCP1, CUBN, CELF2, CUL5, CUL4A, CUX1, CTAGE1, CX3CL1, CX3CR1, CXADR, CLMP, CXCL1P1, CXCL10, CXCL12, CXCL16, CXXC1, CAP1, CGAS, CNGB3, CCNA1, CCNA2, CNNM2, CCNB1, CCNB2, CCNB3, CCNK, DMTF1, CCND1, CCND2, CCND3, CCND3P1, CDK1, CDK11A, CDK11B, CDK13, CDK2AP1, CDKN1A, CDKN1B, CDKN1C, CDKN2A, CDKN2B, CDKN2C, CDKN3, CDKL5, CCNE1, CCNE2, CCNG1, CCNL2, CNTD1, CCNT1, CCNT2, CYLD-AS1, CYLD, LOC106799834, LOC110631417, LOC106780800, CBS, CST9L, CST3, CSRP1, CSRNP3, CHIC2, CRIP3, CSAD, CYSLTR1, CARS1, CTNS, CDA, CMPK1, CYBRD1, CYBA, CYBB, CYBC1, CYB5R3, CYB5A, COA3, COX18, COX20, COX10, COX15, COX4I1, COX4I2, COX5A, COX6A1, COX6A2, COX6B1, COX8A, CYCS, CYC1, CYP3A4, CYP11B2, CYP17A1, CYP19A1, CYP2A13, CYP2C18, CYP20A1, CYP21A1P, CYP21A2, CYP24A1, CYP26A1, CYP27B1, CYP4F22, CYP46A1, CYP51A1, POR, CYGB, CYTH1, CISH, CRLF1, CYFIP2, CLASP1, CKAP4, CYTOR, CIAO2B, CIAO3, CTU1, CTLA4, L2HGDH, DAB1, DACH2, DDB2, DAOA-AS1, DZIP1L, DBH-AS1, DCAF1, DCAF17, DDX1, DDX10, DDX17, DDX20, DDX23, DDX3X, DDX41, DDX54, DDX56, DEAF1, DEL15Q15.3, DHX36, DHX37, DHX8, DAPK1, DAXX, DBR1, DXO, PDSS2, DCN, DOCK8, DOCK10, DOCK11, DEF6, DEFA1, DEFB1, DEFB103A, DEFB103B, DEFB104B, DEFB114, DEFB118, DEFB127, DEFB4A, DHDDS, DHTKD1, DHRS3, DEK, DAZ1, DAZL, DLEU2, DMBT1, DLL1, DLK1, DNER, DTX1, DMTN, DCANP1, DENND11, DENND1B, DENND2B, DTL, DMP1, DSPP, DCK, DGUOK, DHPS, DNASE1, DNASE1L1, DNASE2, DTYMK, DEPDC1B, DEPDC5, ATOD1, ATOD7, DPT, DDU, DES, DSC1, DSG2, DSP,	

	DSTN, DEUP1, DPPA5, DDX60, DDX39A, DDX39B, DHX30, DHX58, DHX9, DGCR8, DIABLO, DGKA, DGKE, DICER1, DKKL1, DKK1, DANCR, DGCR11, DGCR5, DHFR, DBT, DLD, DLAT, DHODH, DPYSL5, DPYD, DUS2, DIP2C-AS1, DPEP1, DPP9, DPP10, DPP6, PPIP5K2, DPH3, DIS3, DIS3L2, DIP2B, DDR2, DLG1, DAAM2, DACT1, DVL1, CLLS2, DMAC1, DLX3, DLC1, DLEC1, DLGAP5, DMXL2, DCLRE1A, DCLRE1C, DDIAS, DDIT4, LIG4, DNMT1, DMAP1, DNMT3A, DNMT3B, DNTT, POLB, POLD1, POLD3, POLH, POLG2, POLG, POLI, POLK, POLL, POLM, POLN, POLQ, PRIM1, DNA2, TOP1, TOP2A, TOP2B, TOPBP1, TOP3B, DNAJA2, DNAJB1, DNAJB13, DNAJC12, DNAJC13, DNAJC14, DNAJC15, DNAJC19, DNAJC21, DNAJC24, DNAJC28, DNAJC3, DNAJC5, DNAJC6, LOC111674472, DND1, DALIR, DOK7, DOLK, DDOST, DPM1, DPM3, DPAGT1, DDC, DCT, DBH, DRD1, DRD2, DRD3, DRD4, DRGX, DOT1L, DUX4, DPF2, DMRT3, DRAIC, DR1, LOC117152611, LOC117152610, DPP9-AS1, DPY19L2, DPYD-AS1, DROSHA, DSCAM-AS1, DST-AS1, DTWD2, DUOX2, DUSP1, DUSP10, DUSP14, DUSP19, DUSP22, DUSP23, DUSP26, DUSP29, DYRK1A, DYRK1B, DYM, DCTN1, DNM1L, DNM2, DYNC2I1, DNAAF11, DNAAF2, DNAH8, DNAH10, DNAI1, DNAL1, DNALI1, DYNC1I2, DNHD1, DYNLL1, DYNLT1, DRC1, DBNDD1, DYSF, DKC1, DST, DTNB, DTNBP1, DAG1, DMD, EP300, E2F1, EMSLR, ELF4, EEA1, EGR1, EBF3, EBNA1BP2, EBP, EVI5, ECSIT, ENC1, EDA, EDAR, EDA2R, ENTPD2, ENPP1, EPG5, EDARADD, EDIL3-DT, EEF1E1-BLOC1S5, EFCAB10, EFCAB12, EFHC1, EFHD2, EFEMP2, EGFL8, EDIL3, EGFR-AS1, EGILA, EGLN1, ELAC2, ELANE, ELN, EMILIN2, ELAVL4, ETFDH, ETFA, ETFB, ERC1, ELOF1, ELL, EFL1, ELP1, ELOC, ELOVL4, EML4, EMB, ETDC, EMD, EMSY, EVL, ENAM, EBLN2, ERVW-1, ERVFRD-1, ERVK3-1, ENG, ENDOV, ERAP1, ERO1A, ERN1, ESM1, EDF1, EPAS1, EDN1, EDNRB, ELMO1, EDC4, EZH2, LOC106694316, ENO2, EVPL, EPX, EID2, EPB41L4A-DT, EPCIP-AS1, EPDR1, EPHA1, EPHA2, EPHA8, EPHB2, EPHB4, EFNA2, EFNA5, EFNB2, EFNB3, EGF, EGFR, EPS15, EPPIN, ECA1, GEFSP7, EREG, EPCAM, ECT2, EMP2, EPHX1, EDEM1, ERLIN2, EMC10, ERBIN, ERBB2, ERCC1, ERCC2, ERCC4, ERCC5, ERCC6L2, ERCC6, ERCC8, ERG28, ERMAP, EPB41, EPB41L1, ERFE, EPO, EPOR, ERAS, ESS2, EME1, ESCO2, ESR1, EBAG9, ESRRB, ETNK1, ETHE1, ETS1, ELK1, ERG, ETV6, ETS1-AS1, ERF, EHMT2, EEF2K, EEFSEC, EEF1A2, EEF1B2, EEF1G, EEF1GP1, EEF2, EIF6, EIF1AX, EIF2AK2, EIF2S1, EIF2S2, EIF2S3, EIF2B1, EIF2B2, EIF2B4, EIF2B5, EIF2B3, EIF3F, EIF4G1, EIF4E, EIF4EBP1, EIF5A, EIF5AL1, ETF1, EWSAT1, EWSR1, EXOC3-AS1, EXOC1, EXOC1L, EXO1, EXD1, EXOSC10, EXOSC5, EXT1, EXTL2P1, EXTL3, XPO1, ESPL1, EVR3, EYA4, EYS, EZHIP, EZR, F11R, F2RL3, F2RL1, FANCM, FANCD2, FAAP100, FAAP24, FIP1L1, FAM111A, FAM161A, FAM181A-AS1, FAM20A, FAM20C, FAM3B, FAM83A-AS1, FAM107A, FAM124A, FAM13A, FAM135A, FAM149B1, FAM163A, FAM167A, FAM168A, FAM170B, FAM177B, FAM184A, FAM216A, FAM225A, FAM30A, FAM53B, FAM76B, FAM81B, FAM83H, FAM89A, FANCD2OS, FUBP1, FDFT1, FAS-AS1, FAIM, FAF2, FADD, FAS, FASLG, FSCN1, FSCN2, FASTKD2, FAT1, FA2H, FAAH, FABP3, FABP12, FABP5P3, FADS1, FASN, FAR1, FAU, FBXL4, FBXW11, FBXW7, FBH1, FBXO11, FBXO28, FBXO3, FBXO32, FBXO43, FBXL19-AS1, FCAR, FCER1A, FCER1G, FCER2, FCGRT, FCGR1A, FCGR2A, FCGR2B, FCGR2C, FCGR3A, FCGR3B, FCRL4, FCHO2, FEB1, FER, FER1L4, FRMD7, FARP1, FDX1, FRRS1L, FTH1, FTL, FTMT, FECH, FES, FATE1, FEZF1, FGD5-AS1, FGR, FHIP1B, FBL, FBN1,	

	FGA, FGB, FGG, FGL2, FGF2, FGF10, FGF11, FGF12, FGF13, FGF14, FGF19, FGF21, FGF23, FGFBP2, FGFR1, FN1, FANK1, FBRS, FSIP1, FCN3, FIGN, FLG, FLNA, FBLIM1, FIBIN, FKBP10, FKBP11, FKBP1A, FKBP1B, FKBP5, FKBP6, FEN1, FLI1, FLII, FIZ1, FMR1NB, FLT3, FLT3LG, FOCAD, FALEC, FOLH1, FOLR1, FOLR2, FSHR, FDCSP, FL1, FLCN, FNIP1, FSTL1, FPGS, FOXA2, FOXG1, FOXI1, FOXI3, FOXJ1, FOXK2, FOXL2, FOXM1, FOXN1, FOXO1, FOXO3, FOXP1, FOXP2, FOXP3, FOXR2, FTCD, FMN1, FNBP1, FMNL2, FPR1, FOSL1, FOS, FHL1, FOXC2-AS1, FOXD2-AS1, FENDRR, FHIT, FMR1, FXN, FZD4, FRZB, FBP2, FRYL, FTO, FTX, FUOM, FUT10, FUT2, FUT9, FKRP, FH, FAH, FAHD2A, FURIN, FUS, FXYD2, FXYD6-FXYD2, FYN, FYCO1, GNL2, GPSM3, GNA11, GNA13, GNAI1, GNAQ, GNAO1, GNAT2, GNB1, GPBAR1, GPR12, GPR143, GPR15, GPR158, GPR18, GPR182, GPR32, GPR35, GPR55, GPR6, GPR65, GPR88, GPR89A, GPRC5A, GRK2, G3BP1, GABARAPL1, GABARAP, GALK1, GALNS, GALM, GALT, GLA, GLB1, GALC, GAL, LGALS3, LGALS3BP, GCASPC, GTSF1, GGN, GABRA1, GABRA2, GABRA3, GABRA5, GABRA6, GABRB1, GABRB2, GABRB3, GABRD, GABRG2, GABRG3, GABBR1, GGCX, GGH, GGACT, GGT1, GGT2P, GGTLC3, GM2A, GDAP1, GJA1, GJB2, GJC2, GAS5-AS1, GAS6-AS1, GSDMD, GHET1, GIP, GAST, GRP, GRPR, GATA2, GATA6-AS1, LOC106627981, LOC106627982, GC, GCN1, GDNF-AS1, GFRA1, GMDS, GMPPB, GSN, GEN1, GTF2B, GTF2E2, GTF2H1, GTF2I, GTF3C5, GPHN, GCNA, GCSAM, GFOD3P, GHRL, GAN, GIHCG, GIMD1, GIMAP1-GIMAP5, GINS1, GIPC2, GLI1, GDNF, GCM1, GFAP, GLIS1, GLMN, GCG, GLP1R, GCGR, GLCCI1, GCK, GCKR, GNE, G6PC3, G6PD, GPI, GANAB, GBA1, GBA3, GUSB, GAD2, GLUD1, GRIA2, GRID2, GRIK1, GRIN1, GRIN2A, GRIN2B, GRIN3A, GRM7, GLUL, GOT2, GPT, GLS, GATD3, GFPT1, QRSL1, QARS1, ENPEP, EPRS1, GATB, EARS2, GLRX5, GCDH, GPX4, GPX8, GSTM1, GSTP1, GSTT1, GSTZ1, GSS, GSR, GAPDH, GPD1, GATM, GLDC, GNMT, GLRA1, GLRA4, GLRB, PYGL, PYGM, GYS1, GSK3B, GYG1, GYPA, GYPC, GP2, GPA33, CGA, GP1BA, GPM6B, GPNMB, GP5, GP6, GPLD1, GLT1D1, GARS1, GLO1, GRHPR, GPC3, GNAS, GARIN5A, GOLM1, GOLPH3, GOSR1, GOLGA3, GOLGA5, GOLGA6L2, GOLGB1, GNRH1, GNRHR, GPANK1, GPKOW, GPATCH2L, GPATCH8, GPC3-AS1, GPN1, GRN, GNLY, GZMB, GAB2, GADD45A, GADD45B, GADD45G, GAS5, GAP43, GDF15, GFI1, GFI1B, GRB2, GFER, GH1, GHITM, GH-LCR, GHR, GHRH, GTF3C2-AS2, GUF1, GTPBP1, GTPBP3, GCH1, GAPVD1, GIMAP2, GAMT, GDA, GBP1, GUCY1A1, GUCY1B1, GUCY2D, GUK1, GET4, GUSBP1, H1-0, H1-10, H1-4, H19, HLX, H2AC4, H2AC11, H2AC12, H2AC13, H2AC14, H2AC15, H2AC16, H2AC17, H2AC18, H2AC19, H2AC20, H2AC21, H2AC25, H2AX, H2BC15, H2BC21, H2BW1, H3C1, H3C12, H3C13, H3-3B, H3-4, H3-7, LOC112533672, LOC112272621, LOC117125594, H4C5, H4C11, H4C12, H4C13, H4C14, H4C15, H4C16, HAND2-AS1, HP, HPR, HRK, HAS2-AS1, LOC106099062, HBS1L, HCGVIII-2, HCK, HAX1, HAND2, HSP90AA1, HSP90AA2P, HSP90B1, HSPA1L, HSPA12A, HSPA13, HSPA14, HSPA1A, HSPA1B, HSPA4, HSPB1, HSPD1, HSPE1, HSF1, HSFX1, HERC5, HERC1, HACE1, HECW1, HHAT, HLTF, HELLS, HCLS1, HPGDS, HEBP1, HMOX1, HBA1, HBB, HBD, HBE1, HBG2, HBQ1, HBZ, HJV, HPX, HPLH1, HEPACAM2, HS6ST2, HSPG2, HS3ST3B1, HS3ST4, HGSNAT, HPSE, HBEGF, HDGF, HEPACAM, HAVCR2, HVBS7, HCCAT5, HEIH, HULC, HGF, HNF4A, HNF4G, HAMP, HEPH, HEPHL1, ENSG00000188078, HEYL, HNRNPA1,	

	HNRNPA2B1, HNRNPK, HNRNPDL, HNRNPH1, HNRNPH2, HK2, HEXA, HEXB, H6PD, HHIPL2, HHLA2, HIC2, HIF1A-AS2, HIGD1A, HIGD1C, HMGA2, HMGB1, HMGN1, HPCA, HPCAL4, HNMT, HRH1, HRH2, HRH3, HRH4, HTN3, HDC, HRG, HINT1, HARS1, HARS2, HM13, HMSD, HDAC9, HDAC10, HTATIP2, HIVEP2, HCG25, HCG26, HCP5, HLA-DQB1-AS1, HLA-F-AS1, HLF, HNF1B, HNF1A-AS1, HNRNPK-AS1, HJURP, HLCS, HOXA10, HOXA13, HOXA2, HOXA5, HOXA7, HOXA9, HOXB9, HOXC13, HOXC4, HOXC5, HOXC6, HOXD13, HFE, HGD, HOOK1, HOPX, HORMAD1, HUNK, HRNR, HCFC1, HCFC2, HOTAIR, HOXA-AS2, HOTTIP, HOTAIRM1, HOXA11-AS, HAGLR, HPS1, LOC100500719, HPS3, HPS4, HPS5, HPS6, HRAS, ENSG00000266919, HSCB, HTR2A-AS1, HTRA1, HTT, HAP1, HIP1, HUS1, HAPLN1, HABP2, HMMR, HAS1, HYAL2, HYCC1, HAO1, HADH, HADHA, HADHB, HAGH, HCAR2, HSD3B2, HSD3BP4, HMBS, HSD11B2, HSD17B8, HSD17B10, HSD17B11, HSD17B13, HCN2, HCN1, HCRT, HCRTR2, HPRT1, HIF1A, HYOU1, ICAM4-AS1, IDS, IFI30, LOC119230225, IFNAR2-IL10RB, IFNG-AS1, IFT122P2, IRAIN, IGF2-AS, IGLON5, IK, IKZF1, LOC107988022, LOC107988021, LOC107988024, LOC107988025, IL12A-AS1, ITK, IL6-AS1, IER3, IER3IP1, IRGC, IRGM, IGBP1, IGES, IGHD, IGHE, IGHG1, IGHG2, IGHM, IGH, IGHV3-21, IGHV3-66, LOC102723407, IGKC, IGK, IGKV1D-33, IGLL1, IGSF3, IPO5, IDO1, ICOS, IVNS1ABP, INHA, INHBA, IBTK, ID2, ING1, IKBKG, IKBKB, IKBKE, INAVA, IMPDH2, ITPA, ITPR1, IMPA2, INPP5D, ITPKC, INS-IGF2, INSM1, INS, INSL6, IGF1, IGF1R, IGF2BP2, IGFBP6, INSR, INSRR, IRS4, INTS8, INTS10, INTS11, ITFG1, IBSP, ILK, ITGAM, ITGA2B, ITGB3, ITGBL1, IHO1, ITIH4, ICAM1, ICAM4, IFNA1, IFNA10, IFNA13, IFNA14, IFNA16, IFNA17, IFNA21, IFNAR1, IFI27, IFI6, IFNB1, IFNE, IFNG, IFI16, IFNGR1, IFI44, IFI44L, IFIT2, IFITM3, IFITM10, IFIH1, IFNK, IFNL3, IFNL4, IFNLR1, IFNW1, IRF3, IRF2BP2, ISG20, ISG20L2, IFN1@, IL1A, IL1B, IL1F10, IL1RAP, IL1RAPL2, IL1RN, IRAK4, IL1RL2, IL1R1, IL10, IL10RA, IL10RB, IL11, IL12RB1, IL12A, IL12B, IL13, IL13RA1, IL15, IL15RA, IL16, IL17RA, IL17A, IL17B, IL17C, IL17D, IL17F, IL18, IL18BP, IL18R1, IL18RAP, IL19, IL6, IL2RA, IL2RB, IL2RG, IL20RB, IL21, IL21R, IL22, IL22RA2, IL23R, IL23A, IL24, IL25, IL26, IL27, IL31, IL31RA, IL32, IL33, IL34, IL36A, IL36B, IL36G, IL36RN, IL37, IL4I1, IL7R, IL6ST, ILF2, ITSN1, ISX, IFT122, IFT56, IFT74, IFT81, INVS, INF2, IVL, IQANK1, IQSEC1, IQUB, IQCB1, IQCN, IQGAP3, IQCA1L, IREB2, ISCA2, IBA57, IRX2-DT, ISG15, ISL1, IAPP, IDH1, IARS1, IARS2, ICMT, IVD, IST1, ITCH, IATPR, ITPRID1, IZUMO4, JAG1, JAGN1, JAK2, JAZF1, JMJD7-PLA2G4B, JCHAIN, JPX, JRK, JRKL, JDP2, JUN, JUNB, JAM3, JSRP1, JPH1, KAZA1, KAZA2, KLK1, KLKB1, KLK10, KLK11, KLK14, KLK2, KPNA2, KPNB1, KANSL1, KCNQ1DN, KCNQ1OT1, KDELR3, KLHDC8B, KEAP1, KLHL7, KLHL24, KLHL31, KLHL33, KLHL40, KBTBD13, KBTBD4, KEL, KRT7, KRT10, KRT12, KRT14, KRT16, KRT18, KRT19, KRT20, KRT222, KRT24, KRT26, KRT37, KRT74, KRT76, KRT8P21, KRT81, KRT83, KRT85, KRT86, KRTAP9-2, KRTAP20-4, KDF1, KHDC1, KHDRBS3, KIAA0319, KIAA0319L, KIAA0586, KCP, KIR3DL1, KIR3DS1, KIR3DP1, KIR2DL3, KIR2DL5A, KIR2DL5B, KIR2DS2, KIR2DP1, KLRC1, KLRC2, KLRC3, KLRC4, KLRD1, KLRG1, KLRK1, KLLN, KDR, KIF11, KIF13A, KIF1A, KIF1B, KIF21A, KIF21B, KIF5B, KIF6, KLC1, KNG1, KISS1R, KITLG, KIT, KLF1, KL, KLB, KMT2E-AS1, KRBOX4, KRBA1, KRAS, KREMEN1, KRTAP5-AS1, KTI12, KXD1, L1CAM, LARP1, LACC1, LALBA, LACTB, LDHC, LDHAL6A, LPO, LTF, LBX1, LAMA5-AS1, LMNA, LBR, LMNB1, LMNB2,	

	LAMA3, LAMB3, LAMC2, LSS, LATS1, LARGE1, LCE2B, LCE3B, LCE3C, LAMTOR2, LCK, LRP1, LRP1B, LRAT, LCAT, MBL2, LGMN, LMOD1, LMLN, LEMD3, LPRS, LPRS6, LEP, LEPR, LEPROT, LEPQTL1, LAP3, LCMT2, LDC1P, LRG1, LGI1, LRMDA, LINGO1, LRRC17, LRRC18, LRRC25, LRRC32, LRRC41, LRRC4C, LRRC56, LRRC63, LRRC73, LRRC8A, LGR5, LRRK2, LGI2, LRIT1, LETM2, LZTR1, LNPEP, LARS1, LARS2, ALL2, ALL1, CLLS1, CLLS5, LUNAR1, LAIR1, LECT2, LILRA2, LILRA6, LILRB1, LILRB2, LILRB3, LTK, LST1, LTA4H, LTB4R, LTC4S, LIF, LIFR, LCORL, LIAT1, LNX2, LDB1, LIMD1, LPP, LMO1, LHX1, LMX1B, LIM2-AS1, LIN28B, LINS1, LAT, LIPA, LIPF, LIPJ, LIPI, LDAH, LPIN2, LCN2, LIAS, LSR, LBP, LPL, LPA, LLGL2, LMBRD1, LNCRNA-ATB, LNCBRM, LONP1, LINC01001, LINC01013, LINC01018, LINC01075, LINC01089, LINC01098, LINC01133, LINC01138, LINC01149, LINC01152, LINC01186, LINC01187, LINC01191, LINC01194, LINC01221, LINC01307, LINC01342, LINC01381, LINC01419, LINC01433, LINC01446, LINC01502, LINC01504, LINC01505, LINC01554, LINC01565, LINC01587, LINC01589, LINC00161, LINC01672, LINC00173, LINC01786, LINC01857, LINC01929, LINC01938, LINC02027, LINC00205, LINC02055, LINC02096, LINC00210, LINC02152, LINC02237, LINC02241, LINC02258, LINC00229, LINC02412, LINC02426, LINC02525, LINC02605, LINC00261, LINC02676, LINC02797, LINC00293, LINC02985, LINC03000, LINC03041, LINC00313, LINC00336, LINC00358, LINC00381, LINC00383, LINC00393, LINC00423, LINC00458, LINC00467, LINC00473, LINC00511, LINC00528, LINC00538, LINC00554, LINC00589, LINC00598, LINC00601, LINC00612, LINC00665, LINC00668, LINC00673, LINC00709, LINC00857, LINC00882, LINC00901, LINC00926, LINC00941, LINC00974, LINC-PINT, LINC-ROR, LORICRIN, LDLR, LDLRAD3, LRBA, LSM1, LSM11, LSM14A, LSM2, LSM4, LNPK, LCAL1, LUCAT1, LHB, LHCGR, LYAR, LY75-CD302, LYL1, LYVE1, LAG3, LY6E, LY6G5B, LY6G6D, LY75, LY9, LY96, LCP2, LSP1, LAX1, LEF1, LTA, LTB, LTBR, LYN, LYRM4, KAT2A, KAT2B, KAT6A, KAT6B, KAT8, KDM1A, KDM2B, KDM3A, KDM3B, KDM5A, KDM5C, KDM5D, KDM6A, KMT2A, KMT2B, KMT2C, KMT2D, KMT5B, LPCAT1, LAMP1, LAPTM4B, LYST, LYZ, LOX, LOXL2, KARS1, WM2, MAEA, MPEG1, MACIR, MIF, MSR1, MST1, MST1R, MROH6, MAF, MAFTRR, MAGEA1, MAGEA3, MAGEA4, MAGEA6, MAGEA9, MAGEA9B, MAGEB4, MAGEC2, MAGED2, MAGED4B, MAGEE1, MAGEL2, MAGT1, MPP2, MFSD11, MFSD12, MFSD14A, MFSD8, HLA-B, HLA-DMA, HLA-DMB, HLA-DOB, HLA-DPA1, HLA-DPB1, HLA-DQA1, HLA-DQB1, HLA-DRA, HLA-DRB1, HLA-DRB6, MIP, MVP, MKRN1, MDH2, ME2, MHS2, MALT1, MGAM, MGAM2, MPI, MRC1, M6PR, MPDU1, MAN1B1, MAN2A1, MANBA, MOGS, MAPKAPK3, MAPKAP1, MAPKAPK5-AS1, MROCKI, MKI67, MARVELD3, MRGPRX1, MRGPRX2, MILR1, MAML1, MEG3, MEG8, MATN1, MEPE, MGP, MMP1, MMP10, MMP11, MMP12, MMP13, MMP14, MMP19, MMP25, MMP26, MXRA8, MGA, MLX, MXI1, MASP2, MCL1, MCM3AP-AS1, MCTS1, MDM2, MDM4, MECOM, MTOR, LOC126805948, LOC126806063, LOC126862464, LOC126862662, LOC126862707, LOC126862864, LOC126862866, LOC126807619, LOC126859807, LOC126859690, LOC126860438, LOC126863274, MED13L, MED17, MED19, MED20, MED25, MED27, MED29, MED8, MDC1, MEF2C-AS2, MEFV, MPIG6B, MATK, M1AP, MNS1, MEIG1, MEIS1, MLANA, MC1R, MRAP, MITF, MLPH, MELTF, MROS, MAGI2, MARCHF8, MBOAT7, MBTPS1,	

	MME, MS4A1, MS4A2, MS4A4A, MEN1, MERTK, MIMT1, MEST, MSLN, MET, MMAB, MTDH, MPPE1, MT1DP, MT1E, MT1G, MT3, MTA1, MALAT1, MTX1, MTHFS, MAT1A, MAT2A, MSRA, METAP2, MARS1, MARS2, MBD4, MBD5, MECP2, MCCC2, MTHFD1, MTHFR, MCEE, MMUT, MSMO1, MTAP, METTL1, METTL13, METTL14, METTL16, METTL2A, METTL3, METTL5, METTL6, MVK, MFSD2A, MICA, MIA2, MIA-RAB4B, MIATNB, MICOS10-NBL1, MIR100, MIR101-1, MIR103A2, MIR106A, MIR106B, MIR107, MIR10A, MIR10B, MIR9-1, MIR1180, MIR1183, MIR1197, MIR1200, MIR1207, MIR122, MIR1233-1, MIR1238, MIR124-1, MIR1245B, MIR1246, MIR1247, MIR1248, MIR1251, MIR125A, MIR125B1, MIR126, MIR1262, MIR1263, MIR1264, MIR1268B, MIR127, MIR1275, MIR128-2, MIR1285-1, MIR1291, MIR129-1, MIR1293, MIR1299, MIR1304, MIR1307, MIR130A, MIR130B, MIR132, MIR133A1, MIR133B, MIR134, MIR135A1, MIR136, MIR137, MIR138-2, MIR139, MIR140, MIR141, MIR142, MIR143, MIR144, MIR145, MIR146A, MIR146B, MIR147A, MIR147B, MIR148A, MIR148B, MIR149, MIR150, MIR151A, MIR152, MIR154, MIR155, MIR15A, MIR15B, MIR16-1, MIR17, MIR181A1, MIR181B1, MIR181C, MIR181D, MIR182, MIR183, MIR184, MIR185, MIR186, MIR187, MIR188, MIR18A, MIR18B, MIR1908, MIR190A, MIR190B, MIR191, MIR1914, MIR192, MIR193A, MIR193B, MIR195, MIR196A2, MIR196B, MIR197, MIR198, MIR199A1, MIR199B, MIR19A, MIR19B1, MIR200A, MIR200B, MIR200C, MIR202, MIR203A, MIR204, MIR205, MIR2052, MIR206, MIR208A, MIR208B, MIR20A, MIR20B, MIR21, MIR210, MIR211, MIR2116, MIR212, MIR214, MIR215, MIR216A, MIR217, MIR218-2, MIR219A1, MIR22, MIR221, MIR222, MIR223, MIR224, MIR23A, MIR23B, MIR24-1, MIR25, MIR26A1, MIR26B, MIR27A, MIR27B, MIR28, MIR296, MIR299, MIR29A, MIR29B2, MIR29C, MIR301A, MIR301B, MIR302D, MIR3074, MIR30A, MIR30B, MIR30C1, MIR30D, MIR30E, MIR31, MIR3120, MIR3125, MIR3128, MIR3140, MIR3155A, MIR3158-1, MIR3173, MIR3175, MIR3178, MIR3180-1, MIR3183, MIR3184, MIR3187, MIR3199-1, MIR32, MIR320A, MIR323A, MIR324, MIR325, MIR326, MIR328, MIR329-1, MIR330, MIR331, MIR335, MIR337, MIR338, MIR339, MIR33A, MIR340, MIR342, MIR345, MIR34A, MIR34B, MIR34C, MIR3529, MIR361, MIR3614, MIR362, MIR363, MIR365A, MIR367, MIR3683, MIR369, MIR3691, MIR370, MIR371A, MIR372, MIR373, MIR374A, MIR374B, MIR375, MIR376A1, MIR376C, MIR377, MIR378A, MIR379, MIR381, MIR382, MIR383, MIR3909, MIR3937, MIR3940, MIR3944, MIR409, MIR412, MIR422A, MIR423, MIR424, MIR425, MIR4260, MIR4263, MIR4286, MIR4288, MIR429, MIR4291, MIR4299, MIR4304, MIR432, MIR4423, MIR4436B1, MIR4443, MIR4465, MIR4492, MIR449B, MIR451A, MIR452, MIR4520-1, MIR4523, MIR4536-1, MIR454, MIR455, MIR4659A, MIR4687, MIR4692, MIR4751, MIR4763, MIR4774, MIR4784, MIR4804, MIR483, MIR485, MIR486-1, MIR487A, MIR487B, MIR489, MIR490, MIR491, MIR493, MIR494, MIR495, MIR497, MIR498, MIR499A, MIR500A, MIR500B, MIR501, MIR502, MIR503, MIR504, MIR505, MIR508, MIR509-1, MIR510, MIR511, MIR512-1, MIR513A1, MIR513B, MIR515-1, MIR517A, MIR517B, MIR518A1, MIR518D, MIR518F, MIR519B, MIR520A, MIR524, MIR525, MIR532, MIR542, MIR545, MIR548AA1, MIR548B, MIR548D1, MIR548G, MIR548H4, MIR548O2, MIR551B, MIR561, MIR562, MIR570, MIR5700, MIR574, MIR576, MIR582, MIR584, MIR587, MIR588, MIR589, MIR590, MIR596, MIR599, MIR605, MIR6069, MIR6080, MIR6086, MIR609, MIR6090, MIR6124, MIR6126, MIR615, MIR621, MIR625, MIR627, MIR628,	

	MIR638, MIR641, MIR6499, MIR652, MIR653, MIR654, MIR655, MIR656, MIR660, MIR663A, MIR663B, MIR664A, MIR668, MIR671, MIR6726, MIR6727, MIR675, MIR676, MIR708, MIR744, MIR767, MIR7977, MIR873, MIR877, MIR885, MIR887, MIR888, MIR889, MIR92A1, MIR92B, MIR93, MIR95, MIR96, MIR98, MIR99A, MIR99B, MIRLET7A1, MIRLET7B, MIRLET7C, MIRLET7D, MIRLET7E, MIRLET7F1, MIRLET7G, MIRLET7I, MSMB, MGST1, MCRS1, MACF1, MARK2, MAP1LC3A, MAP1LC3B, MAP1LC3C, MAP1B, MAP2, MAPRE3, MAPT, MTUS1, MAST3, MAST4, MATCAP2, MDN1, MDK, MID2, MIF-AS1, MGR6, MFGE8, MINDY3, MCM10, MCM5, MSBP1, MIR100HG, MIR155HG, MIR17HG, MIR181A1HG, MIR210HG, MIR22HG, MIR223HG, MIR34AHG, MIR3945HG, MIR4435-2HG, MIR7-3HG, MIR99AHG, MSTO1, MINK1, MTARC2, MAVS, MCU, MTCH1, MICOS13, MIEF2, MTFR1, MPV17, MIPEP, MTFMT, MRPS12, MRPS22, MRPS30, MRPS7, MRM2, MTERF1, MTRF1, MTIF2, MTO1, MT-RNR1, MT-RNR2, MT-ATP6, MT-CYB, MT-CO1, MT-CO2, MT-CO3, MT-ND1, MT-ND4L, MT-TA, MT-TR, MT-TN, MT-TD, MT-TC, MT-TQ, MT-TE, MT-TG, MT-TH, MT-TI, MT-TL2, MT-TL1, MT-TK, MT-TM, MT-TF, MT-TP, MT-TS2, MT-TS1, MT-TT, MT-TW, MT-TY, MT-TV, MFN2, MAPK1, MAPK1IP1L, MAPK10, MAPK14, MAPK8IP3, MAP2K2, MAP3K8, MAP3K14, MAP3K20, MAP4K4, MALINC1, MAD2L2, MZT2A, MKKS, MKRN2OS, MKS1, MLLT1, MLLT10, MLLT11, MLLT3, MLXIPL, MME-AS1, MMP2-AS1, MMS19, MN1, MLC1, MSN, MKX, MOCS1, MON1A, MAOB, MGLL, MORC3, MORN1, MOSPD3, MLN, MOV10L1, MOV10, MPHOSPH9, MPLKIP, MPL, MPND, LOC129391106, LOC129388938, LOC129388419, LOC129389544, LOC129388420, LOC129388421, MPV17L, MSX2, MSL2, MTCO3P1, MTSS1, MUC1, MUC12, MUC16, MUC2, MUC20, MUC3A, MUC5AC, MUC5B, MUC7, MCOLN1, MADCAM1, MFHAS1, MPDZ, MS, MS2, MS4, MUS81, MSI2, MUSK, MRAS, MLH1, MSH5, MUTYH, MVP-DT, MX1, MYB, MAX, MAZ, MYC, MINCR, MYCL, MYCNOS, MYCN, MYCNUT, MTBS1, MYD88, MAG, MOBP, MBP, MOG, MPZ, MYT1L, MDS2, MNDA, MLF1, MYEOV, MPO, MYG1, MIAT, MRTFA, MYOC, MEF2A, MEF2B, MDFI, MDFIC, MYOD1, MYOG, MB, MYOM2, MYBPC3, MYBPH, MYH7, MYH11, MYH14, MYHAS, MYO1C, MYO1E, MYO9A, MYO9B, MYL2, MYL6B, MYLK, MYO5A, MYO5B, MYO7A, MYRIP, MYO19, MYO18B, MSTN, MYOT, MTPN, MTM1, MTMR7, MYOZ2, NAGLU, GNPTG, GNPTAB, NAGPA, NAGS, NANP, NAT2, NAT9, ASAH1, NQO1, NAXD, NAXE, NDUFAF2, NDUFS1, NDUFS2, NDUFS7, NDUFS8, NDUFV1, NDUFV2, NDUFA1, NDUFA11, NDUFA2, NDUFA5, NDUFA6, NDUFA9, NDUFB11, NDUFB4, NDUFB8, NDUFB9, NDUFC2, NDUFS4, NDOR1, NOX1, NOXA1, NOXO1, NAA10, NANOG, NANOS1, NAPSA, NARF-AS1, NACA, NPPB, NPR1, NCR3, NKG7, NAXD-AS1, NBAS, NBL1, NBPF3, NBPF10, NBPF11, NBPF12, NBPF13P, NBPF14, NBPF15, NBPF17P, NBPF18P, NBPF19, NBPF7P, NBPF20, NBPF21P, NBPF22P, NBPF25P, NBPF26, NBR1, NCF4-AS1, NCK1, NCKAP1L, NCKIPSD, NCK1-DT, NDST1, NDP-AS1, NDUFV2-AS1, LOC132090450, NEB, NECTIN4, NEDD4, NELFA, NRAV, NRIR, NEIL1, NLK, NPHP4, NGF, NGFR, NHLH2, NES, NSF, NTNG1, NTNG2, NCAM1, NEU1, NRG1, NEXMIF, NBEAL2, NBEAP1, NBAT1, NHEG1, NBLST4, NFASC, NF1, NEFH, NEFL, NEUROG2, NLGN2, NMB, NMBR, NAV1, NRCAM, NEUROD2, NEGR1, NPAS2, NPTX2, NPTXR, NREP, NPFF, NPSR1, NPY, NPY5R, NRP2, NETO1, NPTN, NTS, NTRK1, NTF3, NCF1, NM, NEXN-AS1, NF2, NFE2L2, NFIA-AS2, NKILA,	

	NFKBIA, NFKBIB, NFKBIE, NFKBIL1, NKRF, NFU1, NGF-AS1, NAB2, NGLY1, NHERF1, NHLRC1, NHS, NBN, NNMT, NMNAT1, NAMPT, NAMPTP2, NEK7, NINL, NIPAL4, NIPA2, NIPBL, NISCH, NOS2, NKX2-1, NKX2-1-AS1, NAIP, NLRC4, NLRP3, NLRP11, NLRP12, NLRP13, NME2, MPG, NMRAL1, NMRAL2P, NMT2, NNT-AS1, NOG, NORAD, NCMAP, NHEJ1, NONO, NCAPG, NCAPH2, NSUN2, NOP53, NDP, LOC111365141, NOTCH2NLA, NOTCH4, NALT1, NOVA1, ENSG00000228778, ENSG00000266708, ENSG00000273486, ENSG00000265413, NPC1, NPHP3-AS1, NPHS1, NPHS2, NPRL2, NPRL3, NPSR1-AS1, NPTN-IT1, NR2F1-AS1, NRAS, NQO2, NSA2, NSMCE3, NAPA, SGSH, NT5C1B-RDH14, NTAN1, NTHL1, NUAK2, NUBPL, NFIX, NFKB1, NFAT5, NFE2, NUMA1, NEAT1, NPIPB8, NPAT, NRBP1, NSD1, NCOA3, NCOR2, NR1I2, NXF1, NFYA, NFYB, NFYC, NXT1, NABP1, NUCB1, NCL, NPM1, NPM3, NUP107, NUP133, NUP160, NUP205, NUP210, NUP214, NUP37, NUP85, NUP93, NUP98, LOC106865369, NTPCR, NAP1L4, NOD2, NUDT10, NUDT16, NUDT6, LOC107126288, LOC107197952, LOC107133509, NUTM1, NYX, MGMT, OARD1, OBSCN, OCA2, OCLN, OCRL, ODAPH, ODAM, OBP2A, OFD1, OGA, OLAH, OLFM1, OMP, OR1F1, OR1F2P, OR10G6, OR2AK2, OR4C16, OR51I1, OR8K3, OLIG2, OPALIN, OCM, OSM, OSMR, ONECUT1, OPA1, OPRD1, OPRM1, OPN1LW, OPN4, OPTN, ORAI1, LOC107133510, LOC107181288, ORC1, ORMDL3, OAT, ODC1, OAZ3, OTC, OFCC1, ORM1, OSGEP, OSGEPL1, OSTF1, OGN, OMS, OTOF, OTOG, OTUD5, OTUD7A, OTULIN, OTULINL, ODF1, ODAD2, OPA3, OVCH1-AS1, OXA1L, OXSR1, OXER1, OXGR1, OSBP2, OSBPL1A, OSBPL7, OXTR, OXT, P2RY8, LOC126805576, LOC126862230, LOC126806757, PRECSIT, PRAL, PAF1, PAX5, PILRA, PHOX2A, PHOX2B, PITX2, PALLD, PALMD, PPT1, PTF1A, PDX1, PNLIP, PANX1, PANK2, PAPPA-AS1, PAPPA, PON1, PTH, PTH1R, PTHLH, PACRG, PACRGL, PRKN, PARK7, PNLDC1, PALB2, PVALB, PASD1, PATL1, PNPLA3, PTCH1, PTCHD4, PEG10, PAUPAR, PAX8-AS1, PXN, PBX2, PSIP1, PCBP2-OT1, PCNA-AS1, PCLAF, PLUT, PDLIM7, PBK, PDZK1, PDZK1IP1, PELI1, PTX3, PEPD, PI15, PI3, PMPCA, PDF, PYY, PGLYRP1, PADI4, PIN1, PPIC, PTRH2, PTRHD1, PRF1, PCM1, PLIN1, PER2, POSTN, PMP2, PMP22, PRPH2, PPL, PXDN, PXD	

Table S4: The information in topology parameters of intersection targets.

Intersection targets	Degree	Betweenness Centrality	Closeness Centrality
TNF	47	0.669354839	0.387608203
PTGS2	35	0.588652482	0.153506842
STAT3	26	0.532051282	0.081417175
NFKB1	25	0.51552795	0.04490009
HDAC1	15	0.48255814	0.027308601
MAOA	15	0.48255814	0.048485995
ADK	14	0.402912621	0.049173071
AR	13	0.466292135	0.051728581
MAOB	13	0.488235294	0.043978476
PTGS1	13	0.456043956	0.015745496
XDH	13	0.479768786	0.031506307
CTSS	11	0.458563536	0.007555966
TERT	11	0.443850267	0.005945048
ADA	10	0.471590909	0.030902705
ADORA2A	10	0.485380117	0.052705576
ALOX5	10	0.451086957	0.011577712
BCHE	10	0.456043956	0.003702923
HDAC4	10	0.451086957	0.004527592
PGR	10	0.453551913	0.005316085
SELE	10	0.463687151	0.012943035
CYSLTR1	9	0.432291667	0.041358703
PDE10A	9	0.357758621	0.01377848
PLG	9	0.461111111	0.017558707
BACE1	8	0.441489362	0.001078016
FASN	8	0.466292135	0.032338993
HDAC3	8	0.387850467	0.002810476
PDE11A	8	0.342975207	0.003571085
PDE2A	8	0.342975207	0.003571085
PDE4A	8	0.451086957	0.062736364
TTR	8	0.432291667	0.008283429
HDAC6	7	0.432291667	0.000692486
NAMPT	7	0.441489362	0.010134233
NOS2	7	0.458563536	0.025292534

PDE4D	7	0.35021097	0.002233265
PDE5A	7	0.34439834	0.014396272
PDE7A	7	0.341563786	0.000593135
PSEN1	7	0.430051813	0.001156316
PTGES	7	0.434554974	0.000440788
AKR1B10	6	0.373873874	0.013779669
ALOX15	6	0.427835052	0
FGFR1	6	0.410891089	0.005421422
GLO1	6	0.368888889	0.033473762
MGLL	6	0.408866995	0.010710953
MIF	6	0.451086957	0.004757889
NOX4	6	0.448648649	0.000274932
P2RY12	6	0.441489362	0.01361499
PLA2G2A	6	0.427835052	0
RARA	6	0.372197309	0.001747904
TYR	6	0.436842105	0.000680104
CNR1	5	0.408866995	0.004617848
CTSK	5	0.417085427	0.000478463
DHFR2	5	0.421319797	0.020870707
GCGR	5	0.427835052	0.018218916
NR4A1	5	0.434554974	0
PDE4B	5	0.453551913	0.01868599
PTAFR	5	0.434554974	0.002251006
PTGES2	5	0.434554974	0.011526569
TKT	5	0.391509434	0.018224511
TRPA1	5	0.427835052	0.003037607
ADORA2B	4	0.365638767	0.003839293
EPHX1	4	0.341563786	0.008696509
INSR	4	0.370535714	0.024295272
OXTR	4	0.408866995	0.003081924
PDE9A	4	0.277591973	0
TBXA2R	4	0.341563786	0.00038068
THRA	4	0.34439834	0.000764992
ADORA3	3	0.351694915	0.001398928

AKR1C1	3	0.294326241	0.002167555
CNR2	3	0.34439834	0.000755636
CTSL	3	0.406862745	0
CYP27B1	3	0.421319797	0.000114278
F5	3	0.334677419	0.001740519
MAP3K8	3	0.419191919	0
CCKAR	2	0.309701493	0
CFD	2	0.408866995	0
PNMT	2	0.336032389	0
TYMP	2	0.32046332	0
AHR	1	0.319230769	0
CCKBR	1	0.302919708	0
HAO1	1	0.270358306	0
PKN1	1	0.319230769	0
PLEC	1	0.402912621	0
PRKCG	1	0.27124183	0
SLC22A12	1	0.325490196	0
TDP1	1	1	0
TOP1MT	1	1	0

Table S5: The information of top 20 signaling pathway.

Pathway	Fold Enrichment	*P*-Value	Genes enrichment in the pathway
Phenylalanine metabolism	22.9	0.0072	MIF, MAOA, MAOB
Arachidonic acid metabolism	14	0.0000088	ALOX15, ALOX5, PLA2G2A, PTGES2, PTGES, PTGS1, PTGS2
Tyrosine metabolism	13.6	0.003	MIF, MAOA, MAOB, PNMT, TYR
Antifolate resistance	12.2	0.024	DHFR2, NFKB1, TNF
Purine metabolism	9.5	0.00000077	ADA, ADK, PDE10A, PDE11A, PDE2A, PDE4A, PDE4B, PDE4D, PDE5A, PDE7A, PDE9A, XDH
Morphine addiction	8	0.00081	PDE10A, PDE11A, PDE2A, PDE4A, PDE4B, PDE4D, PDE7A, PRKCG

Serotonergic synapse	6.4	0.0023	ALOX15, ALOX5, MAOA, MAOB, PTGS1, PTGS2, PRKCG
Leishmaniasis	6.3	0.024	NOS2, NFKB1, PTGS2, TNF
AGE-RAGE signaling pathway in diabetic complications	6.1	0.0086	NOX4, NFKB1, SELE, STAT3, TNF
Parathyroid hormone synthesis, secretion and action	5.8	0.01	CYP27B1, FGFR1, PDE4A, PDE4B, PDE4D, PRKCG
Nucleotide metabolism	5.7	0.031	ADA, ADK, TYMP, XDH
Toxoplasmosis	5.5	0.012	ALOX5, NOS2, NFKB1, STAT3, TNF
TNF signaling pathway	5.4	0.013	CFD, IL1B, IL2, IL6, NFKB1, PRKCA, PRKCB, STAT3
Alcoholism	5.2	0.00077	MAP3K8, NFKB1, PTGS2, SELE, TNF
Calcium signaling pathway	3.9	0.0042	ADORA2A, ADORA2B, CCKAR, CCKBR, CYSLTR1, FGFR1, NOS2, OXTR, PTAFR, PRKCG, TBXA2R
cAMP signaling pathway	3.8	0.0094	ADORA2A, NFKB1, OXTR, PDE10A, PDE4A, PDE4B, PDE4D
Neuroactive ligand-receptor interaction	3.7	0.00067	HDAC1, HDAC3, HDAC4, HDAC6, NFKB1, PRKCG
Viral carcinogenesis	3.6	0.024	HDAC1, HDAC3, HDAC4, HDAC6, NFKB1, STAT3
Metabolic pathways	2.6	0.000000064	ADA, ADK, AKR1B10, AKR1C1, ALOX15, ALOX5, CYP27B1, DHFR2, FASN, GLO1, HAO1, MIF, MAOA, MAOB, MGLL, NAMPT, NOS2, PNMT, PDE10A, PDE11A, PDE2A, PDE4A, PDE4B, PDE4D, PDE5A, PDE7A, PDE9A, PLA2G2A, PTGES2, PTGES, PTGS1, PTGS2, TYMP, TKT, TYR, XDH

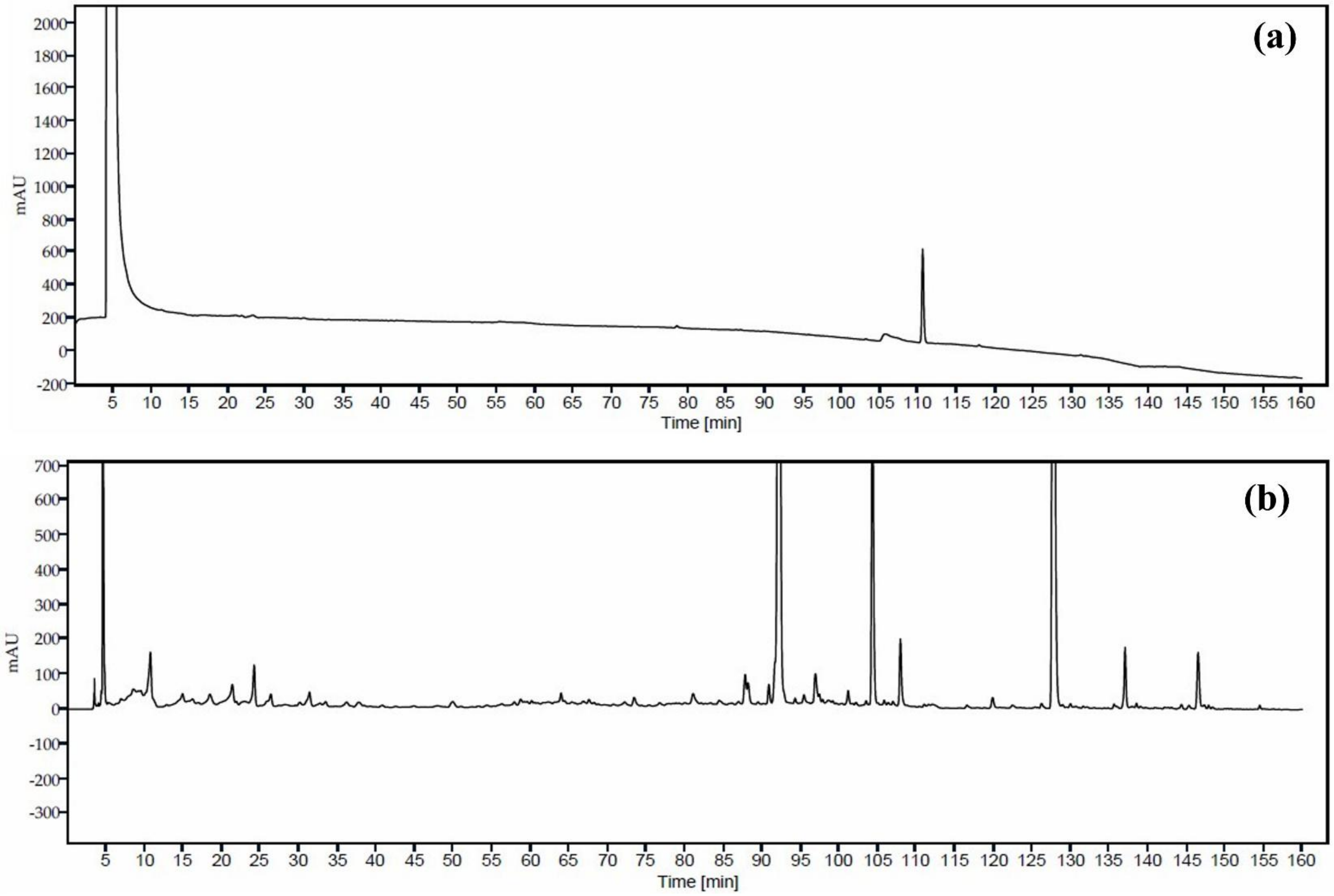

Figure S1. HPLC chromatogram of the obacunone standard recorded at a wavelength of 214 nm (**a**). HPLC chromatogram of BLW extract recorded at a wavelength of 214 nm (**b**).

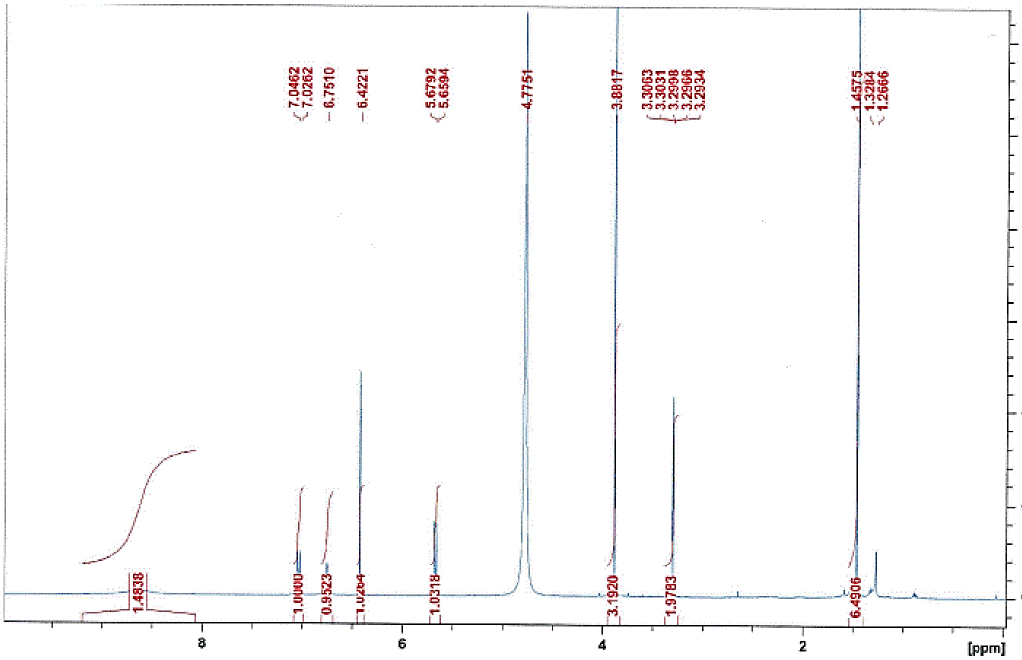

Figure S2. ^{1}H NMR spectrum (400 MHz, CD_3OD) of perforatic acid (**2**).

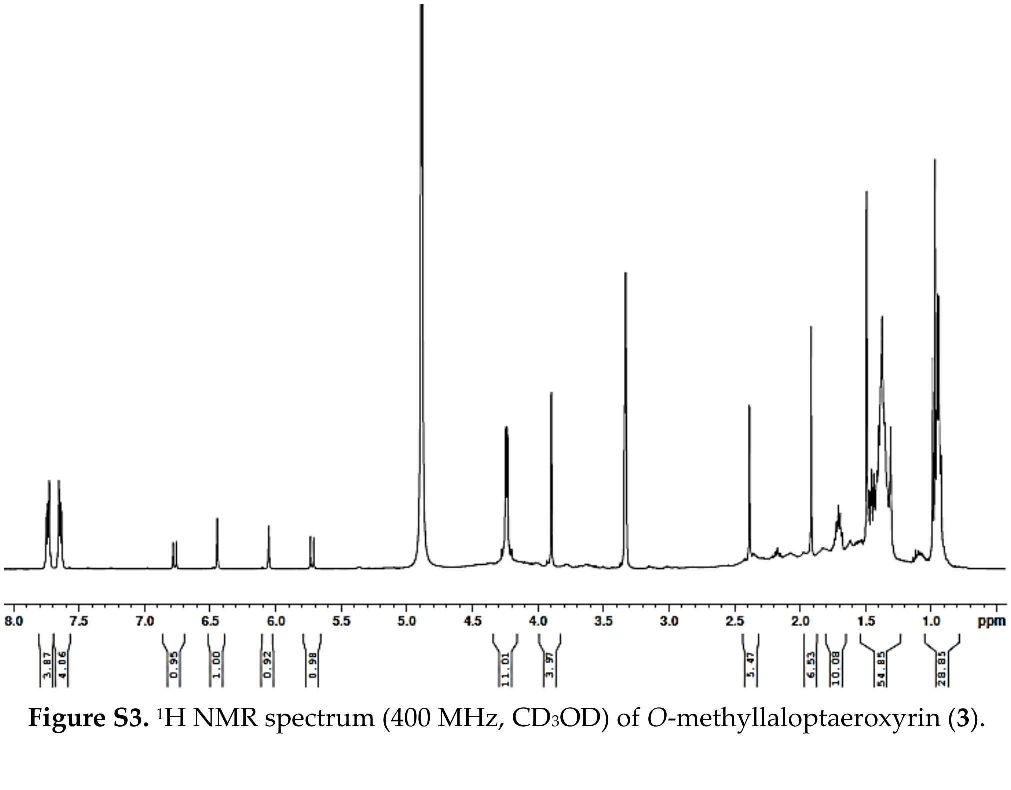

Figure S3. ^{1}H NMR spectrum (400 MHz, CD_3OD) of *O*-methyllaloptaeroxyrin (**3**).

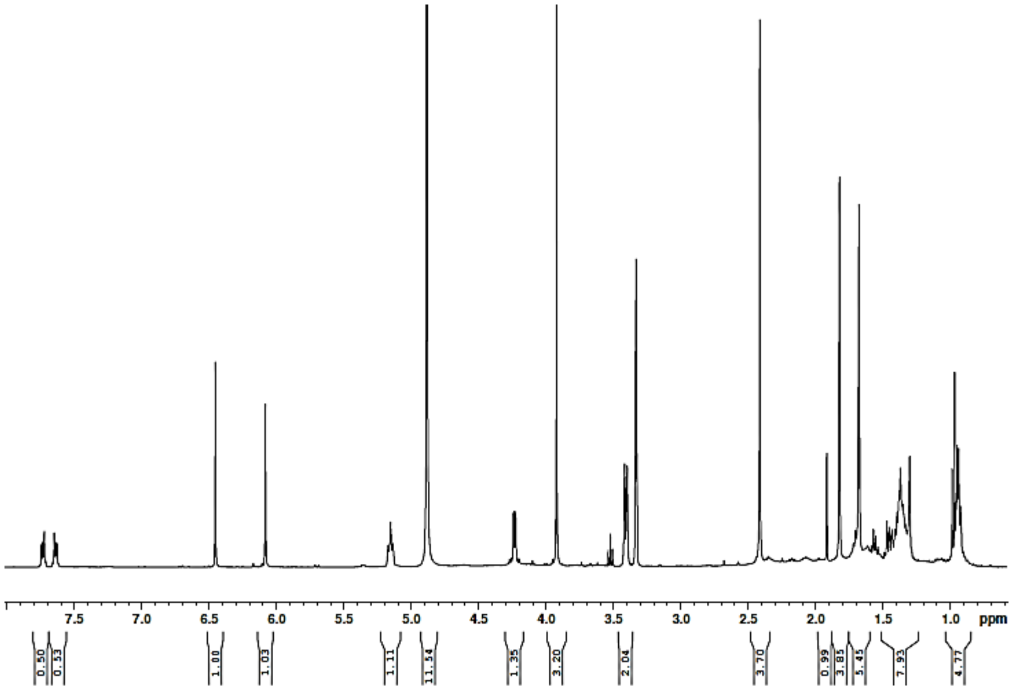

Figure S4. ^{1}H NMR spectrum (400 MHz, CD_3OD) of peucenin-7-methyl ether (**5**)
